# Lnc‐CHRM4‐2:1 Inhibits M2 Polarization and Efferocytosis of Macrophages by Downregulating MerTK and SLC2A1 in Rheumatoid Arthritis

**DOI:** 10.1155/jimr/1718207

**Published:** 2026-02-27

**Authors:** Jinjin Chu, Jie Zang, Lili Zhang, Zhuojian Qu, Haibo Li, Chunjuan Yang, Jiamei Sun, Linlin Gai, Xuecheng Sun, Donghua Xu

**Affiliations:** ^1^ Medical Research Center, Weifang People’s Hospital, Shandong Second Medical University, Weifang, 261000, China, wfph.cn; ^2^ School of Basic Medicine Sciences, Shandong Second Medical University, Weifang, 261000, China; ^3^ Department of Rheumatology and Immunology, Weifang People’s Hospital, Shandong Second Medical University, Weifang, 261000, China, wfph.cn; ^4^ Department of Traumatic Orthopedics, Weifang People’s Hospital, Shandong Second Medical University, Weifang, 261000, China, wfph.cn

**Keywords:** efferocytosis, long noncoding RNA, MerTK, rheumatoid arthritis, SLC2A1

## Abstract

**Objective:**

Rheumatoid arthritis (RA) is a common chronic and systemic autoimmune disease. Long noncoding RNAs (lncRNAs) have been documented to play important roles in the pathogenesis of RA. This study is aimed to investigate the differentially expressed lncRNAs in RA and explore the underlying roles and mechanisms of RA‐specific lncRNAs.

**Methods:**

Peripheral blood mononuclear cells (PBMCs) from three RA patients and three healthy controls were detected by transcriptome sequencing analysis. Enrichment analysis was performed to identify the potential functional categories and signal pathways. The expressions of the screened dysregulated lncRNAs in RA were further validated using quantitative real‐time polymerase chain reaction (qRT‐PCR). The effects of the candidate lncRNA lnc‐CHRM4‐2:1 on the proliferation and apoptosis of Raw264.7 cells were determined by cell counting kit‐8 (CCK‐8), 5‐ethynyl‐2′‐deoxyuridine (EdU) proliferation assays, and Annexin V‐APC/PI staining flow cytometry assay. qRT‐PCR and western blot were performed to detect the expression of functional molecules related to M1/M2 macrophage polarization and efferocytosis.

**Results:**

The expression of lnc‐CHRM4‐2:1 was significantly higher in PBMCs of RA patients compared to healthy controls. Lnc‐CHRM4‐2:1 was negatively associated with the level of serum CCP of RA patients, suggesting that it was a RA‐specific lncRNA. Overexpression of lnc‐CHRM4‐2:1 could potentially affect cell proliferation and apoptosis, promote M1 polarization, inhibit M2 polarization, and the mRNA and protein expressions of MerTK and SLC2A1 in Raw264.7 cells.

**Conclusion:**

Lnc‐CHRM4‐2:1 is an RA‐specific lncRNA, which inhibits macrophage M2 polarization and efferocytosis by downregulating MerTK and SLC2A1. Lnc‐CHRM4‐2:1 may be considered a potential diagnostic biomarker and therapeutic target for RA.

## 1. Introduction

Rheumatoid arthritis (RA) is a common chronic and systemic autoimmune disease characterized by symmetrical polyarticular synovitis, articular cartilage, and bone destruction, eventually leading to joint injury and disability. In addition to joints, multiple organs and systems can be affected in RA patients, causing systemic complications. RA is most common in women, with a global incidence of 0.5%−1.0% and the onset age between 35 and 50 years old [1]. The etiology of RA is complicated, including genetics, environment, and smoking [2, 3]. The pathogenesis of RA remains unclear. Currently, the diagnosis of RA is mainly based on clinical symptoms, signs, laboratory examination, and imaging examination. More effective diagnostic strategies are warranted because of easily missed diagnosis of early, atypical, and inactive RA patients. Furthermore, although disease‐modifying antirheumatic drugs (DMARDs) and immunosuppressants can partially alleviate the progression of RA, about one‐third of RA patients fail to achieve sustained clinical remission [4, 5]. Therefore, elucidating the pathogenesis of RA and screening promising therapeutic targets remain the focus of attention in the field of RA research.

Long noncoding RNA (lncRNA) is a class of noncoding RNA with the length of more than 200 nucleotides [6]. LncRNAs are involved in the regulation of gene expression and various biological processes (BPs) such as genomic imprinting, transcriptional regulation, epigenetic regulation, chromosome conformation, and cell cycle regulation [7, 8]. A number of studies have found that the abnormal expression of lncRNAs broadly participated in the pathogenesis of cancer, cardiovascular diseases, and immune diseases [7, 9–11]. Increasing studies have shown that lncRNAs play important roles in the pathogenesis of RA by regulating innate and adaptive immune responses [12–17]. However, the mechanisms of lncRNAs in regulating the phenotype and function of disease‐specific immune cell populations in RA, especially macrophages, are largely unknown.

Efferocytosis refers to the process of the clearance of apoptotic cells by macrophages and other phagocytes, which is a key mechanism for the body to maintain homeostasis [5, 18]. Defects in the clearance of apoptotic cells caused by impaired efferocytosis exacerbate RA [19]. C‐mer tyrosine kinase (MerTK) and solute carrier family 2 member 1 (SLC2A1) have been documented to be key regulatory molecules involved in regulating macrophage efferocytosis [20, 21]. MerTK is a member of the TAM receptor tyrosine kinase family, playing a key role in the anti‐inflammatory feedback regulation. It also mediates macrophage efferocytosis and exerts a protective role in RA [4]. SLC2A1 is also a critical marker for efferocytosis. Nevertheless, the precise roles and mechanisms of MerTK and SLC2A1 in regulating macrophage function and macrophage efferocytosis in RA remain unclear. Moreover, lncRNAs involved in MerTK‐ and SLC2A1‐mediated efferocytosis in RA have rarely been reported.

In this study, the transcriptome sequencing technology is applied to screen the differential expression profile of lncRNAs in peripheral blood mononuclear cells (PBMCs) from RA patients. We further explore the role and mechanism of RA‐specific lncRNA in regulating macrophage phenotype and function in RA through a series of cellular and molecular experiments to elucidate the pathogenesis and identify new biomarkers for the diagnosis and treatment of RA.

## 2. Materials and Methods

### 2.1. Patients and Controls

A total of 40 initially diagnosed and untreated RA patients registered in our hospital from February 2022 to April 2022 were recruited, and 30 age‐ and sex‐matched healthy subjects enrolled for physical examination during the same period participated in the present study. All RA patients met the diagnostic criteria of RA developed by the American Society of Rheumatology/European Anti‐Rheumatic Alliance in 2009. This study was approved by the Ethics Committee of Weifang People’s Hospital, Shandong Second Medical University (2020YX009). All participants’ informed consent was obtained before enrollment in the study. For the transcriptome RNA‐sequencing (RNA‐seq) analysis, PBMCs extracted from three initially diagnosed and untreated RA patients and three sex‐ and age‐matched healthy controls (two females and one male, mean age 50.7 years old and 51 years old, respectively) were used. The sample size for the initial RNA‐seq screening (*n* = 3 per group) was selected based on established conventions in the field, which are powered to identify major differentially expressed genes (DEGs) [22–24]. For further validation, the level of the differentially expressed lncRNAs screened by RNA‐seq was verified by quantitative real‐time polymerase chain reaction (qRT‐PCR) of PBMC samples from 36 RA patients and 21 healthy controls. In the RA group, there were 8 males and 28 females, ranging from 21 to 70 years old, with an average age of 52.1 years old. The healthy control group (NC group) included 9 males and 12 females, ranging from 26 to 71 years old, with an average age of 45.7 years old. The general clinical data of RA patients and healthy controls are shown in Table 1.

**Table 1:** General characteristics of RA patients and healthy controls.

Characteristics	RA (*n* = 36)	NC (*n* = 21)	*p*‐Value
Age, mean years (range)	52.1 (21–70)	45.7 (26–71)	0.082
Gender, female/male	8/28	9/12	0.101
PLT (10^9^/L)	264.06 (69.49)	236.81 (50.83)	0.123
RF (IU/mL)	217.27 (330.54)	N/A	—
CCP (U/mL)	538.25 (650.26)	N/A	—
ESR (mm/h)	20.29 (18.03)	N/A	—
CRP (mg/L)	7.80 (9.23)	N/A	—

*Note*: Average values or numbers of each group are shown. Standard deviations are shown in parentheses. CCP, anti‐cyclic citrullinated peptide antibody.

Abbreviations: CRP, C‐reactive protein; ESR, erythrocyte sedimentation rate; N/A, not available; PLT, platelet; RF, rheumatoid factor.

### 2.2. Blood Samples Isolation

Peripheral blood samples (2 mL) were obtained from RA patients and healthy controls. The samples were collected in ethylene diamine tetraacetic acid (EDTA) tubes. PBMCs were isolated using Ficoll density gradient centrifugation (TBD, Tianjin, China) according to the protocol and stored at −80°C for further experiments.

### 2.3. Transcriptome RNA‐seq

Total RNAs were extracted from three initially diagnosed and untreated RA patients and three sex‐ and age‐matched healthy controls for whole transcriptome sequencing. RNA extraction, whole transcriptome sequencing, and data analysis of all samples were completed by Shanghai OE Biomedical Technology Co., Ltd. (Shanghai, China). In brief, total RNAs were first extracted from the samples, and an RNA library was constructed. Next, high‐throughput sequencing was performed using the Illumina sequencing platform. Then, DESeq software was used to standardize the count number of lncRNAs in each sample, and the basemean value was used to estimate the expression level. The multiple difference was calculated using a negative binomial distribution test. The screening criteria of all DEGs were as follows: fold change (FC) > 2 and *p*‐value or FDR (adjusted *p*‐value) < 0.05. All raw data were deposited in the database of Genome Sequence Archive for Human (GSA‐Human, HRA007699).

### 2.4. Geneontology (GO) and Kyoto Encyclopedia of Genes and Genomes (KEGG) Pathway Analysis

According to GO function annotation information of lncRNA‐derived genes, GO function enrichment analysis of differentially expressed lncRNAs was conducted. The hypergeometric distribution test was used to calculate the significance of lncRNA enrichment in each GO function set. Pathway analysis of differentially expressed lncRNAs was performed using the KEGG database, and the significance of differentially expressed lncRNA enrichment in each pathway entry was calculated by the hypergeometric distribution test.

### 2.5. qRT‐PCR

Total RNAs were extracted using TRIzol reagent (Invitrogen, Carlsbad, USA) and reverse‐transcribed into cDNA using HiScript II Q RT SuperMix for qPCR reagent (Vazyme Biotech, Nanjing, China). Differentially expressed lncRNAs were verified by qRT‐PCR with Taq Pro Universal SYBR qPCR Master Mix (Vazyme Biotech, Nanjing, China). This experiment was performed on the BioRad CFX96 Real‐Time PCR System (BioRad, CA, USA). The primers were designed by online PrimerBank and synthesized by Sangon Biotech (Sangon, Shanghai, China). Their sequences are shown in Table 2. The PCR conditions were as follows: initial denaturation at 95°C for 5 min; 45 cycles of denaturation at 95°C for 30 s, followed by combined annealing/extension at 60°C for 1 min. GAPDH was used as an internal control to normalize lncRNA expression by calculating 2^−△△Ct^.

**Table 2:** Primer sequences.

Gene	Primer sequence (5’–3’)	Product length (bp)
*Lnc-CHRM4-2:1*	Forward GAAGGAACAATTACCTTCATCTGC	243
	Reverse CACGACCAAATCCGCCTCTA	
*Lnc-SLFN5-1:4*	Forward CTAGCCCAGACTTCAGGAGGA	105
	Reverse ATGCAGTGGGTCCAAGCAA	
*Lnc-NBPF19-5:1*	Forward ACTCCGCATTACACCACTGA	192
	Reverse CCCGAAACAGTACCAGGCAA	
*Lnc-ZNF253-2:7*	Forward TGGGAGAGGACAAGCTACCA	96
	Reverse GCCGCAACTTTGGAAGTCAG	
*Lnc-BCL2A1-3:2*	Forward CAGAGGGTAAGGCAAGGCTG	108
	Reverse AGTGCCTGAGTGCAACTGTC	
*TCONS_00027971*	Forward GCAACAAGGAAGGTGGTCAAG	90
	Reverse CCCAGATCCTCCATCCAACAC	
*Lnc-IL1B-2:1*	Forward GCCCTTTCCTGTCCCATTGA	148
	Reverse TCTTGCCCACCTGTGATGTC	
*Lnc-ARHGAP29-7:1*	Forward TCCAAAAGGGCTGGATTGCT	124
	Reverse AGGTCTGCTTTGCCAGTACG	
*Human GAPDH*	Forward GGAGCGAGATCCCTCCAAAAT	197
	Reverse GGCTGTTGTCATACTTCTCATGG	
*Mouse GAPDH*	Forward AGGTCGGTGTGAACGGATTTG	123
	Reverse TGTAGACCATGTAGTTGAGGTCA	
*Mouse IL-6*	Forward CTGCAAGAGACTTCCATCCAG	131
	Reverse AGTGGTATAGACAGGTCTGTTGG	
*Mouse TNF-α*	Forward CTGAACTTCGGGGTGATCGG	122
	Reverse GGCTTGTCACTCGAATTTTGAGA	
*Mouse iNOS*	Forward ACATCGACCCGTCCACAGTAT	177
	Reverse CAGAGGGGTAGGCTTGTCTC	
*Mouse CD206*	Forward CTCTGTTCAGCTATTGGACGC	132
	Reverse CGGAATTTCTGGGATTCAGCTTC	
*Mouse Arg1*	Forward CTCCAAGCCAAAGTCCTTAGAG	185
	Reverse AGGAGCTGTCATTAGGGACATC	
*Mouse IL-10*	Forward CTTACTGACTGGCATGAGGATCA	101
	Reverse GCAGCTCTAGGAGCATGTGG	
*Mouse MERTK*	Forward GAAGGAGAGTTTGGGTCTGTAA	93
	Reverse GTTGTCCAACTTCATGGTCTTC	
*Mouse SLC2A1*	Forward CAGTTCGGCTATAACACTGGTG	156
	Reverse GCCCCCGACAGAGAAGATG	
*Mouse SLC16A1*	Forward GGTGGGCAGTGTTAGTCGG	170
	Reverse GATAGGACCTCCAGCATACATGA	
*Mouse MAF*	Forward GGAGACCGACCGCATCATC	173
	Reverse TCATCCAGTAGTAGTCTTCCAGG	
*Mouse SLC7A11*	Forward AGGGCATACTCCAGAACACG	160
	Reverse GGACCAAAGACCTCCAGAATG	
*Mouse GDF15*	Forward CTGGCAATGCCTGAACAACG	142
	Reverse GGTCGGGACTTGGTTCTGAG	
*Mouse-MafB*	Forward TTCGACCTTCTCAAGTTCGACG	179
	Reverse TCGAGATGGGTCTTCGGTTCA	

*Note:* MAF, v‐maf avian musculoaponeurotic fibrosarcoma oncogene homolog, C‐MAF; MafB, V‐maf musculoaponeurotic fibrosarcoma oncogene family, protein B.

Abbreviations: Arg1, arginase 1; CD206, cluster of differentiation 206; GAPDH, glyceraldehyde‐3‐phosphate dehydrogenase; GDF15, growth differentiation factor 15; IL‐6, interleukin‐6; IL‐10, interleukin 10; iNOS, inducible nitric oxide synthase; SLC16A1, solute carrier family 16 member 1; SLC7A11, solute carrier family 7 member 11; TNF‐α, tumor necrosis factor α.

### 2.6. The Construction of Competitive Endogenous RNA (ceRNA) Regulatory Network

The miRNA target prediction software miRanda was used to predict the target miRNA and miRNA‐target (miRNA–mRNA or miRNA–lncRNA) relationship pairs (miRanda filtering conditions: Max Score ≥ 140 or Max Energy ≤ −10). Then, the regulatory relationship between lncRNA–mRNA was predicted according to the shared miRNAs. The predicted ceRNA relationship was visualized by Cytoscape software to construct the lncRNA–miRNA–mRNA network.

### 2.7. Cell Culture

Mouse Raw264.7 cells (GeneChem, Shanghai, China) were cultured in the incubator with 5% CO_2_ at 37°C, with Dulbecco’s modified Eagle’s medium (DMEM, Gibco, NY, USA) added with 10% fetal bovine serum (FBS, Gibco, NY, USA) and 1% penicillin/streptomycin (Invitrogen, Carlsbad, USA). Cells in the logarithmic growth phase were used for subsequent experiments. Raw264.7 cells were stimulated to polarize into proinflammatory M1 macrophages using 100 ng/mL LPS and 20 ng/mL IFN‐γ. About 20 ng/mL IL‐4 was used to stimulate Raw264.7 cells into anti‐inflammatory M2 macrophages. Cells were cultured at 37°C and 5% CO_2_ for 6 h and 12 h for subsequent experiments, respectively.

### 2.8. Cell Transfection

Lentivirus vector construction and lentivirus packaging were completed by OBiO Technology Co., Ltd. (OBiO, Shanghai, China). Raw264.7 cells were divided into the control group and lnc‐CHRM4‐2:1‐overexpressed group. Cells in the control group were transfected with empty lentivirus vector, and cells in the lnc‐CHRM4‐2:1‐overexpressed group were transfected with lnc‐CHRM4‐2:1‐overexpressed lentivirus vector. Stable cell lines were obtained by puromycin screening, named LV‐NC and LV‐lnc‐CHRM4‐2:1, respectively. The mRNA expression of lnc‐CHRM4‐2:1, CD11c, IL‐6, TNF‐α, iNOS, CD163, CD206, Arg‐1, and IL‐10 in the M0/M1/M2 polarization state of LV‐NC and LV‐lnc‐CHRM4‐2:1 cell lines was detected by qRT‐PCR. The primer sequences are shown in Table 2.

### 2.9. Cell Counting Kit‐8 (CCK‐8) Assay

The stably transfected cells were incubated in a 96‐well plate according to the density of 1500 cells/well. After 3 h of culture, the serum‐free medium was changed overnight, and the DMEM containing 10% FBS was replaced the next day, marked as 0 day. After 0, 1, 2, 3, and 4 days of cell culture, 10 μL CCK‐8 (DojinDo, Shanghai, China) solution was added to each well and incubated for 2 h. The OD value was detected at the wavelength of 450 nm.

### 2.10. 5‐Ethynyl‐2′‐Deoxyuridine (EdU) Agentia Assay

The stably transfected cells were incubated in a 96‐well plate according to the density of 3 × 10^4^ cells/well. After 3 h of culture, the serum‐free medium was changed overnight, and the DMEM containing 50 μM EdU (RIBOBIO, Guangzhou, China) and 10% FBS was replaced and incubated for 6 h. Then, cells were fixed with 4% paraformaldehyde for 30 min and neutralized for 5 min with 2 mg/mL glycine. After 0.5% Triton X‐100 treatment for 10 min, cells were incubated for 30 min with Apollo dye and Hoechst 33342 dye at room temperature. EdU‐positive cells were evaluated by a Leica inverted fluorescence microscope (Leica, DMi8, Germany). The percentage of EdU‐positive cells was calculated by dividing the number of EdU‐positive cells (red) by the total number of cells stained with Hoechst 33342 (blue).

### 2.11. Cell Apoptosis

The stably transfected cells were incubated in a 24‐well plate according to the density of 2.5 × 10^5^ cells/well. After 3 h of culture, the serum‐free medium was changed overnight, and the DMEM containing 10% FBS was replaced and incubated for 8 h. The H_2_O_2_ treatment group was treated with 0.5 mM H_2_O_2_ (Sigma‐Aldrich, Shanghai, China) to induce apoptosis for 8 h. Then, the cells were collected and washed with PBS buffer. An amount of 5 μL Annexin V‐APC and 10 μL PI (MULTI SCIENCES, Hangzhou, China) was added and incubated in the dark for 5 min. Fluorescence was detected by flow cytometry (Weimi Bio‐Tech, Wmini5268, Guangzhou, China). Early apoptotic cells were labeled with Annexin V‐positive/PI‐negative, and late apoptotic cells were labeled with Annexin V‐positive/PI‐positive. The apoptosis rate was the sum of early and late apoptotic rates.

### 2.12. Western Blot Assay

The total proteins were extracted and separated by 10% SDS–PAGE and were transferred to polyvinylidene fluoride (PVDF) membranes (Millipore, Billerica, MA, USA) with an electrotransfer device. The membranes were blocked using 5% nonfat milk and incubated with iNOS rabbit polyclonal antibody (1:1000, Proteintech Group, Chicago, USA), Arginase‐1 mouse monoclonal antibody (1:5000, Proteintech Group, Chicago, USA), Mertk monoclonal antibody (1:1000, Invitrogen, Carlsbad, USA), SLC2A1 monoclonal antibody (1:1000, Proteintech Group, Chicago, USA), and GAPDH monoclonal antibody (1:5000, Proteintech Group, Chicago, USA). The incubated membranes were incubated with HRP‐conjugated secondary antibodies. Proteins were visualized by ECL (Proteintech Group, Chicago, USA). ImageJ software was used to analyze the gray value of the band. The ratio of the gray value of the target band to GAPDH was used as the relative expression level of the target protein.

### 2.13. Assessment of Apoptosis Induction

Jurkat T cells (GeneChem, Shanghai, China) were cultured in complete RPMI 1640 medium (Gibco, NY, USA) in the incubator with 5% CO_2_ at 37°C. To induce apoptosis, the cells were exposed to UV light (254 nm) for 0, 10, 20, or 30 min, and then incubated for 3 h under the same conditions. Apoptosis was evaluated by staining with Annexin V‐APC and PI, followed by flow cytometric analysis.

### 2.14. Efferocytosis Assay

Jurkat T cells at a density of 10^6^/mL were stained with PKH26 (Umibio, Shanghai, China) for 10 min, followed by two washes. The labeled cells were induced to undergo apoptosis as described previously. Subsequently, these apoptotic Jurkat T cells were cocultured with LV‐NC and LV‐lnc‐CHRM4‐2:1 Raw264.7 cells prestained with Hoechst 33342 (RIBOBIO, Guangzhou, China) at a 1:1 ratio for 2 h, respectively. The efficiency of phagocytosis was quantified by flow cytometry.

### 2.15. Statistical Analysis

GraphPad Prism 9 software was used for data statistical analysis. All experiments were repeated at least three times. Data were presented as the mean ± standard deviation (SD). Two groups of counting data (%) were tested using the chi‐square test. Two groups of measurement data were tested by normal distribution and homogeneity of variance. An unpaired *t*‐test was applied to compare the data of normal distribution and homogeneity of variance between groups, and Welch’s test was used when the variance was uneven. The Mann–Whitney test was used to compare the nonnormal distribution data between groups. One‐way ANOVA was used to compare multiple groups. An ordinary one‐way ANOVA was performed for data with normal distribution and equal variances, and Welch’s test was performed with unequal variances. The Kruskal–Wallis test was performed for nonparametric data. Spearman correlation analysis was used to evaluate the correlation between lncRNA expression levels and clinical features. *p*  < 0.05 was considered to indicate statistical significance.

## 3. Results

### 3.1. Screening Differentially Expressed LncRNAs in RA

The complete transcriptome sequencing of six samples was performed in this study. The distribution of Q30 bases was 95.37%–95.85%, the average GC content was 48.08%, and the genome alignment rate was 96.36%–98.79%. There were 628 differentially expressed lncRNAs in the RA group, including 151 upregulated genes and 477 downregulated genes (Figure 1A,B). The top 20 significantly up‐ and downregulated lncRNAs are shown in Table 3.

Figure 1Analysis of lncRNA expression in PBMCs between RA patients and healthy controls (FC > 2, *p* < 0.05). (A) Differential expression cluster analysis map. Samples are given by column and differentially expressed lncRNAs (adjusted *p* < 0.05) by row, with red as high and blue as low. (B) Volcano map. Log_2_ (fold change) is shown on the *x*‐axis and −log_10_ (*p*‐value) on the *y*‐axis.

**Figure:**
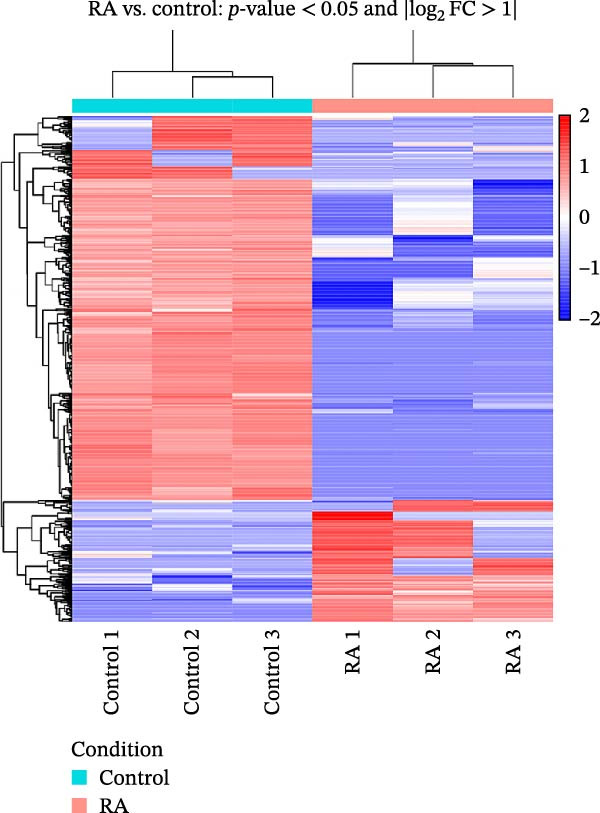
(A)

**Figure:**
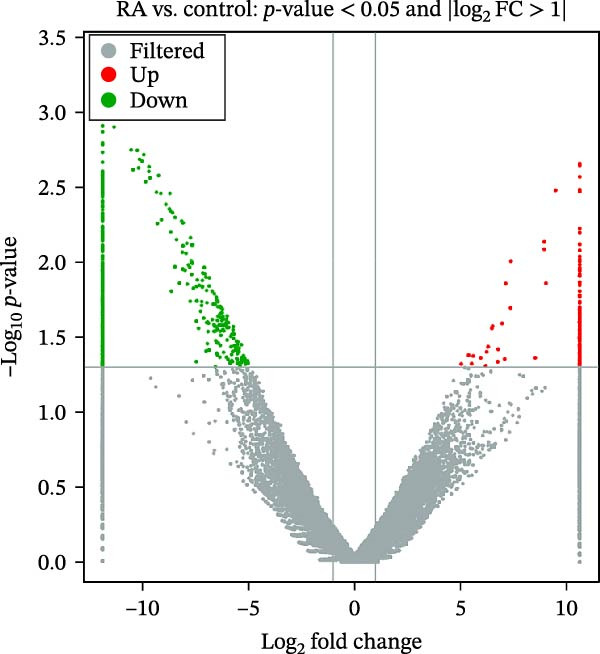
(B)

**Table 3:** Upregulated/downregulated expression of the top 20 lncRNAs in PBMCs of RA patients.

LncRNA_id	Log_2_ (fold change)	*p* Value	Up_down
Lnc‐RCC1L‐4:1	10.6333	0.0022	Up
Lnc‐CHRM4‐2:1	9.4936	0.0033	Up
Lnc‐TRIM69‐3:1	9.0437	0.0138	Up
Lnc‐MON2‐2:5	8.9579	0.0082	Up
Lnc‐KRTAP9‐7‐5:5	8.9547	0.0073	Up
Lnc‐SLFN5‐1:4	8.535	0.0432	Up
Lnc‐FBXO28‐1:12	7.3887	0.0098	Up
Lnc‐SFT2D3‐2:7	7.3704	0.0201	Up
LINC01184:49	7.1398	0.0139	Up
Lnc‐STON2‐5:1	7.0938	0.0441	Up
Lnc‐IGFBP1‐1:2	6.9739	0.0255	Up
Lnc‐TLL1‐4:3	6.7799	0.038	Up
Lnc‐OR4F29‐1:11	6.7591	0.046	Up
Lnc‐NBPF19‐5:1	6.5137	0.0267	Up
Lnc‐BTNL8‐6:2	6.4828	0.0275	Up
Lnc‐PSPC1‐1:5	6.3081	0.0366	Up
LINC02397:27	6.2491	0.0396	Up
Lnc‐ERVW‐1‐3:1	6.1964	0.049	Up
Lnc‐ZNF253‐2:7	5.9617	0.0432	Up
Lnc‐SNAPC3‐5:1	5.6047	0.0422	Up
Lnc‐PERP‐2:32	−11.8941	0.0008	Down
Lnc‐C12orf74‐2:1	−11.7077	0.0006	Down
Lnc‐SERAC1‐7:2	−11.3459	0.0012	Down
Lnc‐TSEN15‐6:1	−10.5415	0.0018	Down
TCONS_00027971	−10.4529	0.0024	Down
Lnc‐ARHGAP29‐7:1	−10.2584	0.0018	Down
Lnc‐GPR137B‐10:1	−10.1896	0.0023	Down
MIR155HG:11	−10.1494	0.002	Down
Lnc‐IL1B‐2:1	−10.0266	0.0021	Down
Lnc‐ITGB8‐13:1	−9.9484	0.0019	Down
Lnc‐FAM207A‐4:1	−9.8637	0.0029	Down
Lnc‐BCL2A1‐3:2	−9.7069	0.0025	Down
Lnc‐ABCA5‐14:2	−9.6961	0.0023	Down
Lnc‐ITGA6‐3:1	−9.6491	0.0027	Down
Lnc‐FAM43A‐13:1	−9.3451	0.0034	Down
Lnc‐SERAC1‐7:1	−9.3061	0.0055	Down
TCONS_00022522	−9.248	0.0026	Down
Lnc‐DNASE1‐8:1	−9.1417	0.0035	Down
Lnc‐CXCR6‐3:1	−9.112	0.0052	Down
Lnc‐IL37‐5:1	−8.9058	0.0041	Down

### 3.2. Functional Analysis of Differentially Expressed LncRNAs

We performed GO enrichment analysis of differentially expressed lncRNAs in PBMCs between RA patients and healthy controls from three aspects: BP, molecular function (MF), and cellular component (CC) (Figure 2A–D). The functional enrichment results showed that differentially expressed lncRNAs were significantly enriched in 765 BP entries, 204 MF entries, and 120 CC items. Differentially expressed lncRNAs were mainly involved in oleic acid response, adiponectin activation signal pathway, long‐chain fatty acid metabolism, linolenic acid metabolism, ganglioside metabolism, mitochondrial outer membrane, peroxisome membrane, mitochondrial ribosome, and MFs, such as decanoic acid‐coenzyme A ligase activity and long‐chain fatty acid‐coenzyme A ligase activity.

Figure 2Enrichment analysis of differentially expressed lncRNAs. (A) GO function BP enrichment analysis of top 20. (B) GO function MF enrichment and analysis of top 20. (C) GO function CC enrichment analysis of top 20. The name of GO entry is represented by *y*‐axis, the enrichment score is expressed by *x*‐axis, the size of *p*‐value is represented by the color of points, and the number of differential lncRNAs covered by each entry is expressed by the size of points. (D) GO functional enrichment analysis (total) top 30. *x*‐axis is the name of the GO entry and *y*‐axis is −log_10_ (*p*‐value). (E) KEGG enrichment analysis of top 20 bubble diagram. *x*‐axis is enrichment score and *y*‐axis is KEGG enrichment result. (F) The distribution map of differentially expressed lncRNAs at KEGG Level 2. *x*‐axis is the percentage of differentially expressed lncRNAs annotated to each Level 2 metabolic pathway to the total number of differentially expressed lncRNAs annotated to KEGG pathway, and *y*‐axis is the name of Level 2 pathway.

**Figure:**
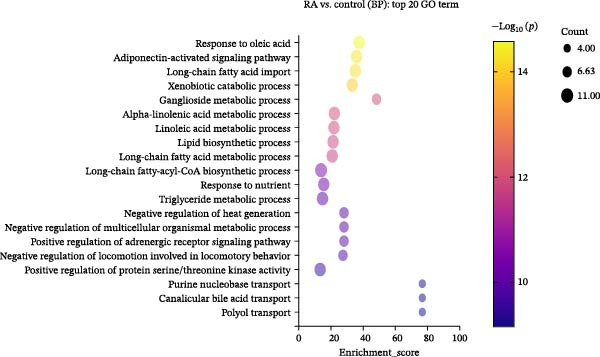
(A)

**Figure:**
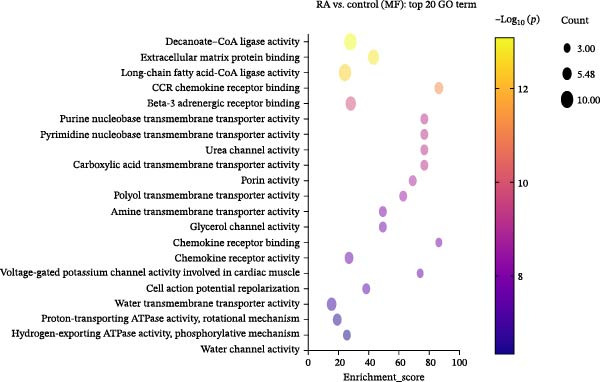
(B)

**Figure:**
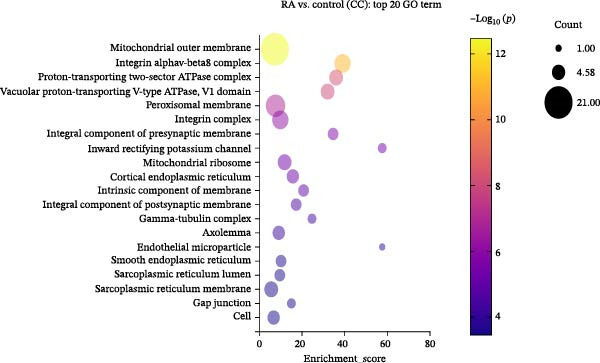
(C)

**Figure:**
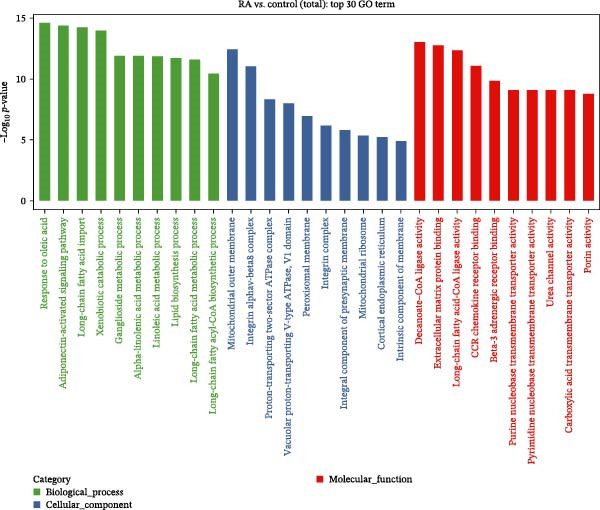
(D)

**Figure:**
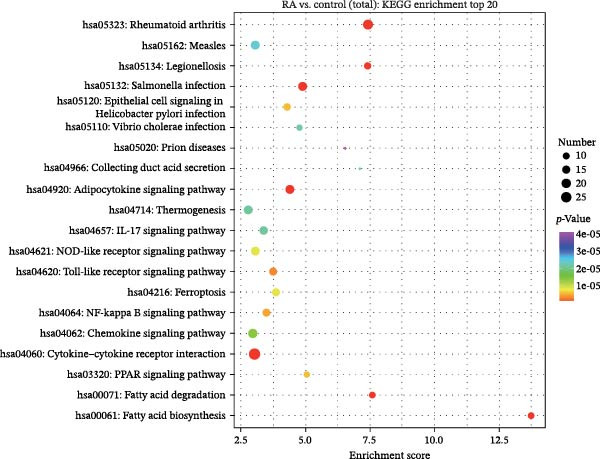
(E)

**Figure:**
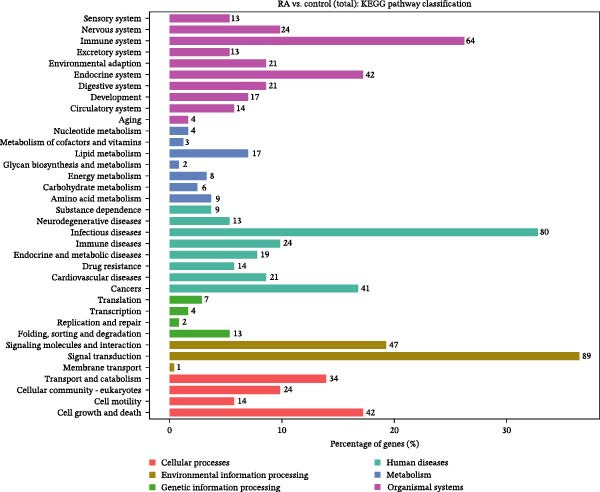
(F)

KEGG pathway analysis showed that 244 differentially expressed lncRNAs were located in 242 KEGG signal pathways, of which 75 pathways were significantly enriched. Among them, RA, Legionellosis, fatty acid biosynthesis/degradation, and cytokine–cytokine receptor interaction signal pathway enrichment were the most significant (Figure 2E,F). RA (ID: hsa05323), cytokine–cytokine receptor interaction (ID: hsa04060), and fatty acid biosynthesis/degradation (IDs: hsa00061 and hsa00071) separately involved 21, 26, 18, and 10 differentially expressed lncRNAs, indicating that differentially expressed lncRNAs may affect the occurrence and development of RA through a variety of immune‐related pathways and inflammatory cytokine signaling pathways.

### 3.3. Verification of Differentially Expressed LncRNAs and Their Correlations With Clinical Data of RA Patients

The upregulated lncRNAs (lnc‐CHRM4‐2:1, lnc‐SLFN5‐1:4, lnc‐NBPF19‐5:1, and lnc‐ZNF253‐2:7) and downregulated lcnRNAs (lnc‐BCL2A1‐3:2, TCONS_00027971, lnc‐IL1B‐2:1, and lnc‐ARHGAP29‐7:1) with larger values of FC in PBMCs of RA patients were verified by qRT‐PCR (Figure 3). The results showed that compared with the NC group, the expressions of lnc‐CHRM4‐2:1, lnc‐SLFN5‐1:4, lnc‐NBPF19‐5:1, and lnc‐ZNF253‐2:7 increased in the RA group (*p*<0.0001, *p*  < 0.0001, *p*  < 0.01, *p*  < 0.05), while the expressions of lnc‐BCL2A1‐3:2, TCONS_00027971, and lnc‐ARHGAP29‐7:1 decreased in the RA group (*p*<0.05, *p*  < 0.05, *p*  > 0.05). These results were consistent with the transcriptional sequencing. The expression of lnc‐IL1B‐2:1 was slightly decreased in RA, and the difference was not statistically significant. Correlation analysis showed that the expression of lnc‐CHRM4‐2:1 was negatively correlated with the level of serum CCP in RA patients (*p*<0.05) but not with the levels of PLT, RF, CRP, and ESR (all *p*  > 0.05) (Figure 4). It suggested that lnc‐CHRM4‐2:1 might be involved in regulating specific immune disorders in RA disease but barely affects the disease activity.

Figure 3qRT‐PCR validation of differentially expressed lnc‐CHRM4‐2:1 in RA (A), lnc‐SLFN5‐1:4 (B), lnc‐NBPF19‐5:1 (C), lnc‐ZNF253‐2:7 (D), lnc‐BCL2A1‐3:2 (E), TCONS_00027971 (F), lnc‐IL1B‐2:1 (G), and lnc‐ARHGAP29‐7:1 (H). Data were expressed as the mean ± SD. (A) Data were analyzed using the Mann–Whitney test; data in (H) were compared by Welch’s test, while data in (B, C, D, E, F, and G) were compared by an unpaired *t*‐test ( ^∗∗∗^: *p* < 0.001;  ^∗∗^: *p* < 0.01;  ^∗^: *p* < 0.05; ns: not significant). Each spot represents a single person.

**Figure:**
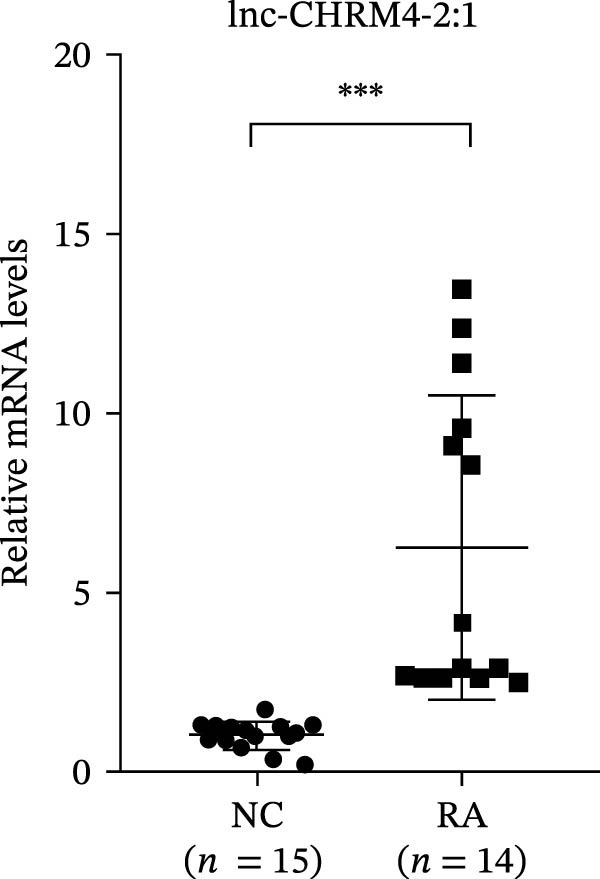
(A)

**Figure:**
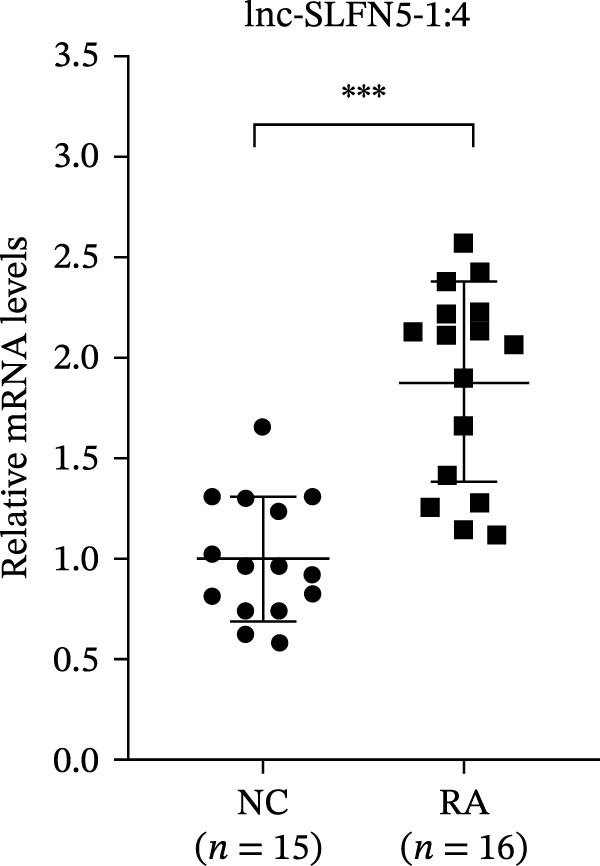
(B)

**Figure:**
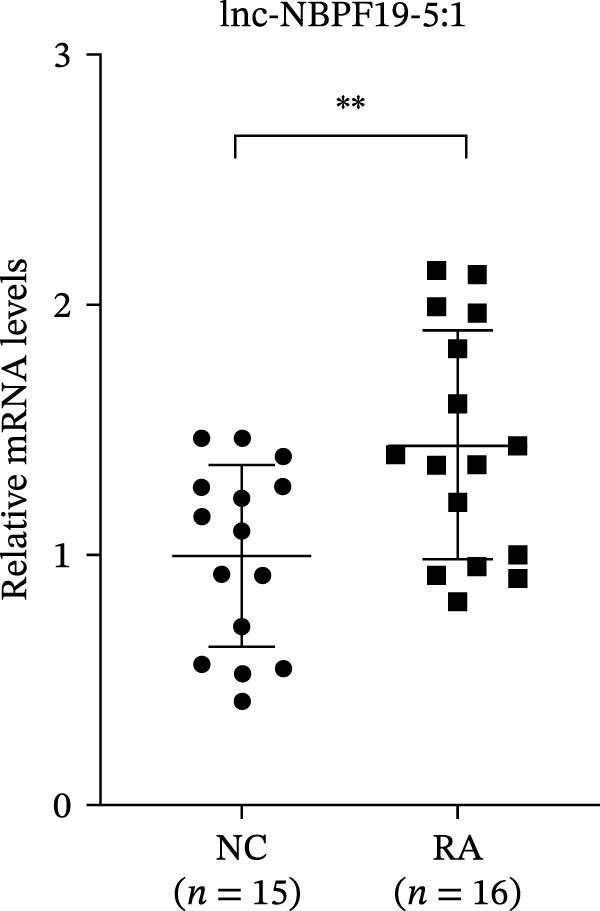
(C)

**Figure:**
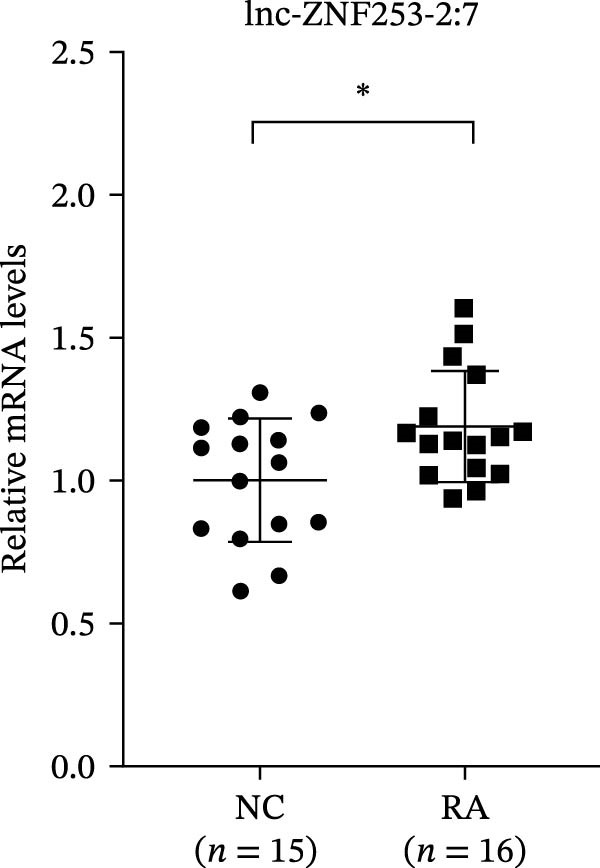
(D)

**Figure:**
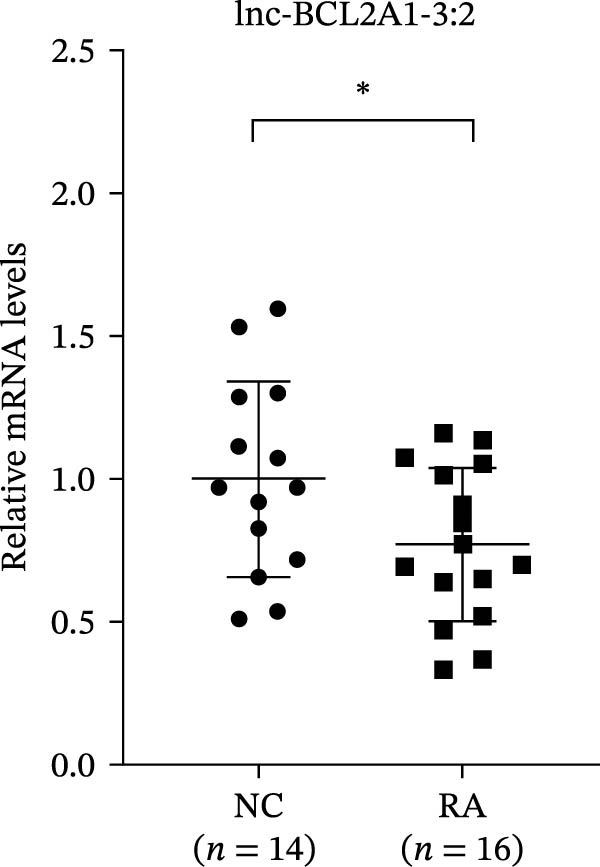
(E)

**Figure:**
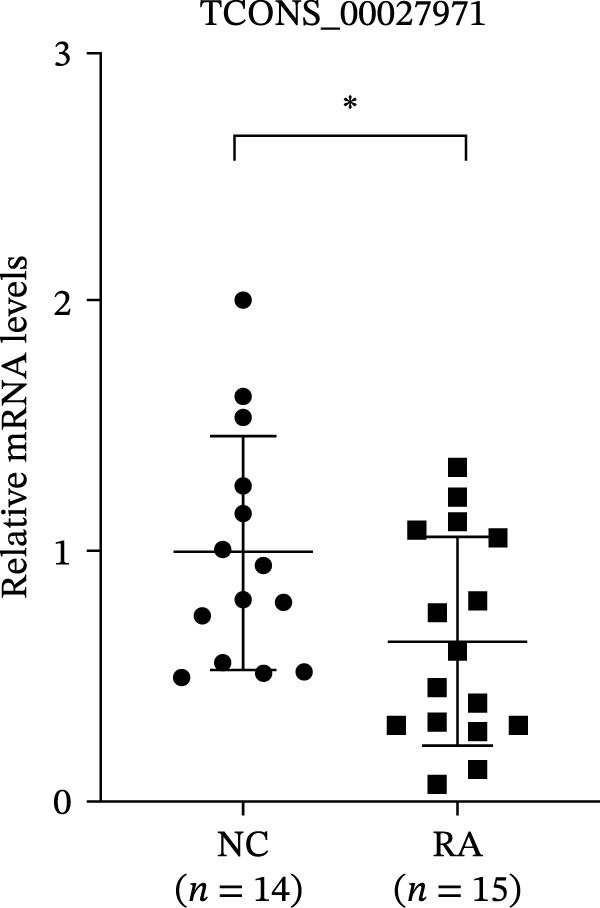
(F)

**Figure:**
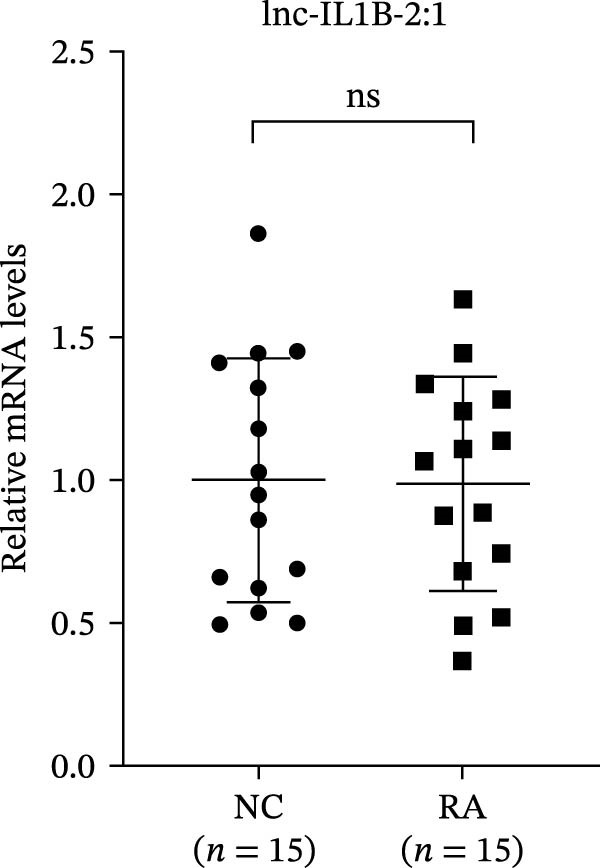
(G)

**Figure:**
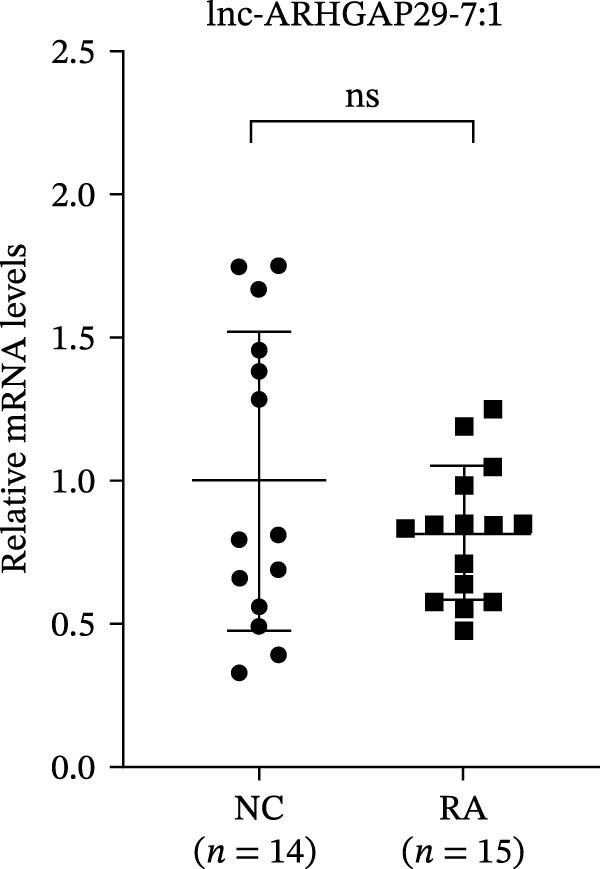
(H)

Figure 4Relationship between the expression of lnc‐CHRM4‐2:1 and clinical indexes of RA patients (A–E), correlation analysis between lnc‐CHRM4‐2:1 and PLT (*n* = 19), RF (*n* = 21), CRP (*n* = 17), ESR (*n* = 17), and CCP (*n* = 13).

**Figure:**
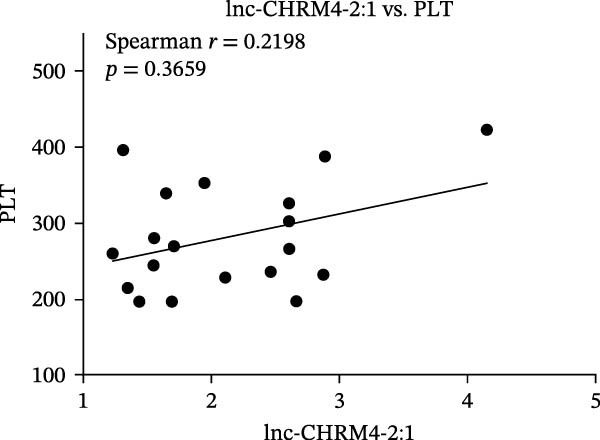
(A)

**Figure:**
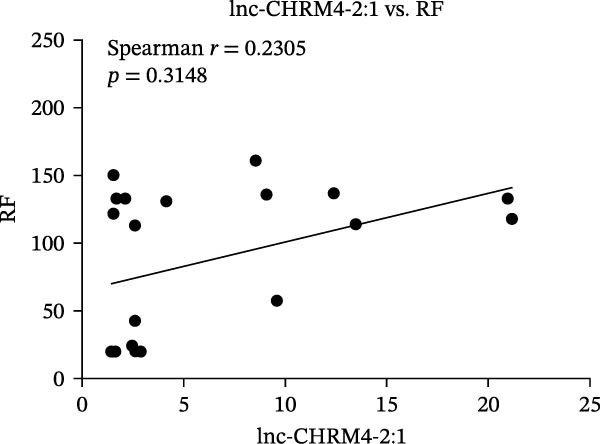
(B)

**Figure:**
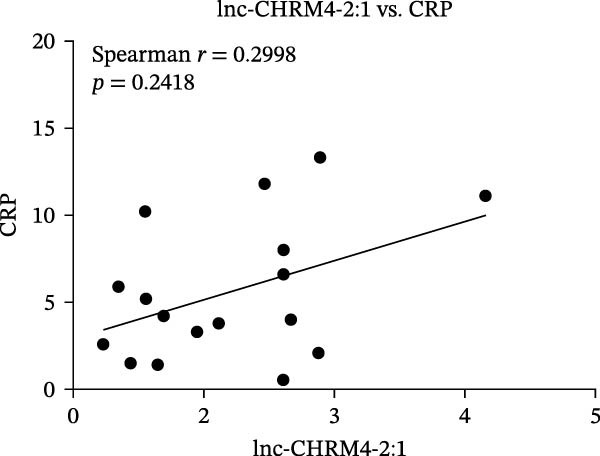
(C)

**Figure:**
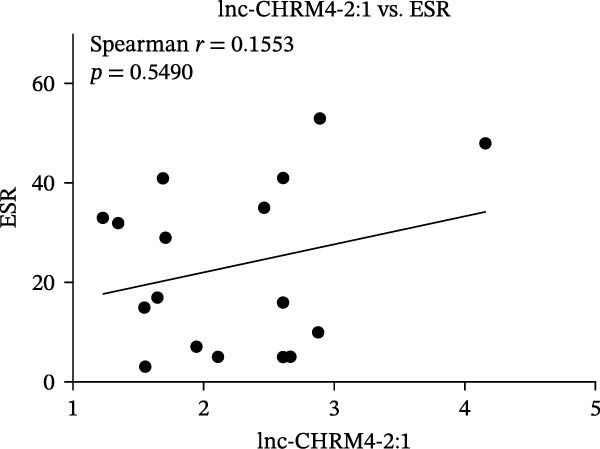
(D)

**Figure:**
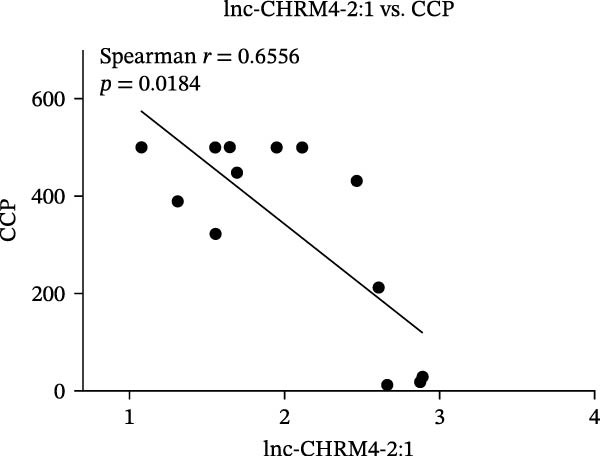
(E)

### 3.4. Construction of Functional Gene Network for Potential Regulation of Lnc‐CHRM4‐2:1

**Figure 5:**
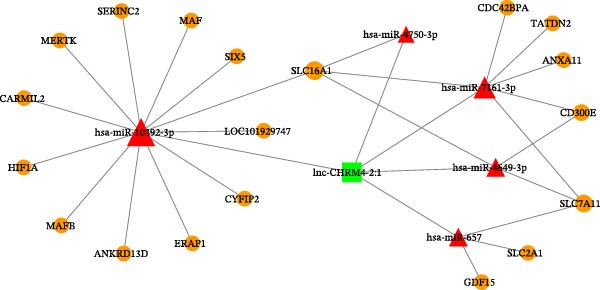
Constructing lnc‐CHRM4‐2:1‐miRNA–mRNA ceRNA regulatory network.

Based on the predicted results of miRanda, we found that there were five potential target miRNAs for lnc‐CHRM4‐2:1, including hsa‐miR‐10392‐3p, hsa‐miR‐7161‐3p, hsa‐miR‐4750‐3p, hsa‐miR‐657, and hsa‐miR‐4649‐3p. The ceRNA network of lnc‐CHRM4‐2:1‐miRNA–mRNA was built (Figure 5). The results implicated that lnc‐CHRM4‐2:1 potentially targeted SIX5, CYFIP2, SERINC2, CARMIL2, ANKRD13D, LOC101929747, ERAP1, TATDN2, CDC42BPA, ANXA11, MerTK, HIF1A, MafB, MAF, SLC16A1, SLC2A1, SLC7A11, CD300E, and GDF15 by negatively regulating hsa‐miR‐10392‐3p, hsa‐miR‐7161‐3p, hsa‐miR‐4750‐3p, hsa‐miR‐657, and hsa‐miR‐4649‐3p. According to the targeted mRNAs, we hypothesized that lnc‐CHRM4‐2:1 might affect macrophage efferocytosis by regulating efferocytosis‐related factors of MerTK and SLC2A1 in RA. To further clarify this hypothesis, we carried out the following cellular and molecular experiments in vitro.

### 3.5. Construction of the Lnc‐CHRM4‐2:1‐Overexpressed Raw264.7 Cell Line by Lentivirus Transfection

The expression of lnc‐CHRM4‐2:1 in LV‐NC and LV‐lnc‐CHRM4‐2:1 groups was detected by qRT‐PCR. Significantly increased expression of lnc‐CHRM4‐2:1 was observed in the LV‐lnc‐CHRM4‐2:1 group compared with the LV‐NC group (Figure 6A). The EGFP fluorescence under an inverted fluorescence microscope showed a good transfection efficiency (Figure 6B).

Figure 6Effect of lnc‐CHRM4‐2:1 on the proliferation and apoptosis of Raw264.7 cells. (A) Expression of lnc‐CHRM4‐2:1 verified by qRT‐PCR. Data were expressed as the mean ± SD. (A) Data were compared by Welch’s test (*n* = 3,  ^∗∗^: *p* < 0.01). (B) The transfection efficiency of lnc‐CHRM4‐2:1‐overexpressed lentiviral vector in Raw264.7 cells by fluorescence microscope. (C) Overexpression of lnc‐CHRM4‐2:1 on the growth of Raw264.7 cells was examined by CCK‐8 assay. (D, E) Overexpression of lnc‐CHRM4‐2:1 on the growth of Raw264.7 cells was examined by EdU assay. Data were expressed as the mean ± SD (*n* = 3,  ^∗^, *p* < 0.05; ns, not significant). Scale bar: 50 μm. (F) The apoptosis rate was detected by flow cytometry and Annexin V‐PI double staining. (G) Calculation of the apoptosis rate (early apoptosis rate plus late apoptosis rate). Data were expressed as the mean ± SD. (B) Data were compared by ordinary one‐way ANOVA (*n* = 3,  ^∗∗∗^: *p* < 0.001;  ^∗∗^: *p* < 0.01).

**Figure:**
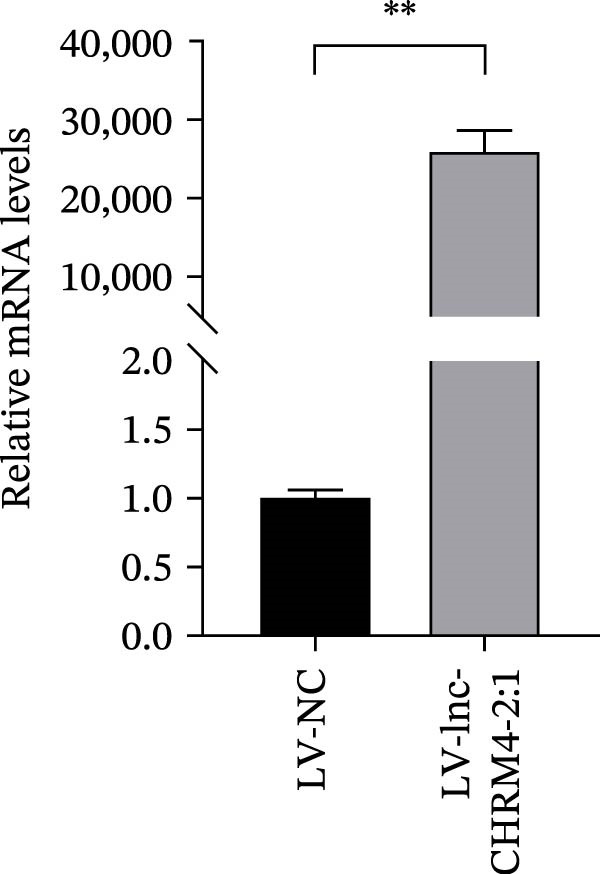
(A)

**Figure:**
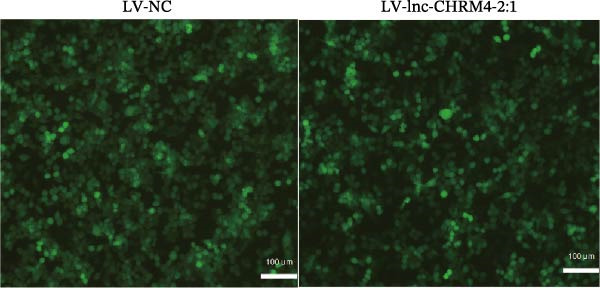
(B)

**Figure:**
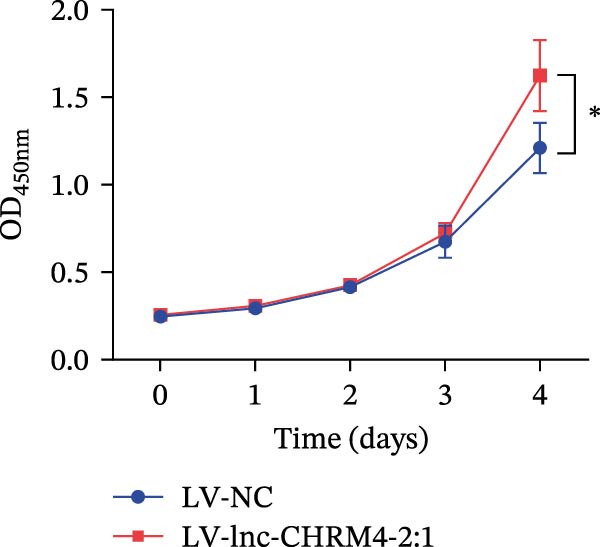
(C)

**Figure:**
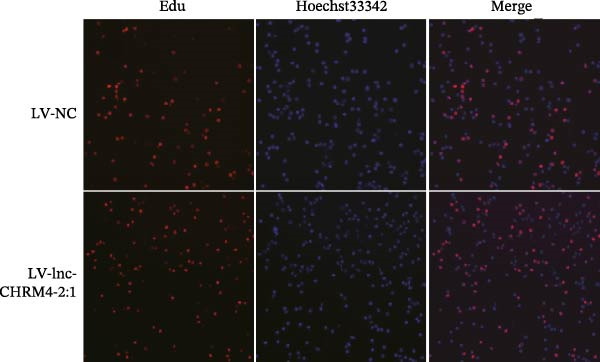
(D)

**Figure:**
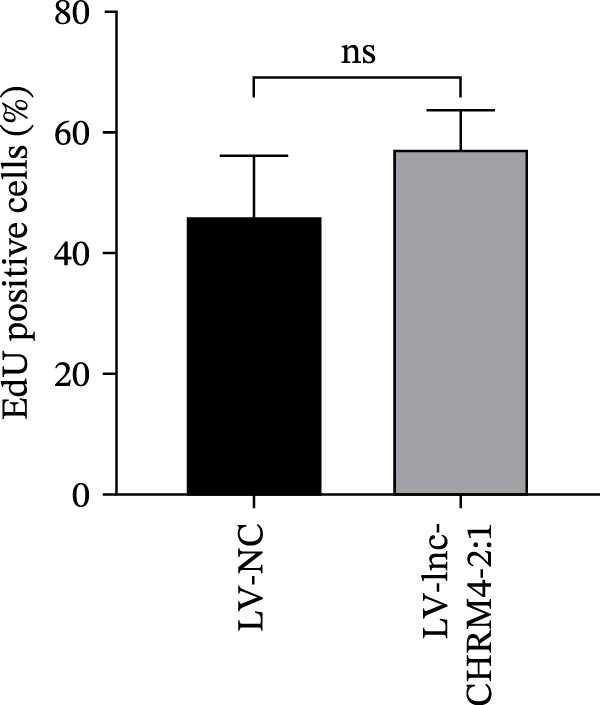
(E)

**Figure:**
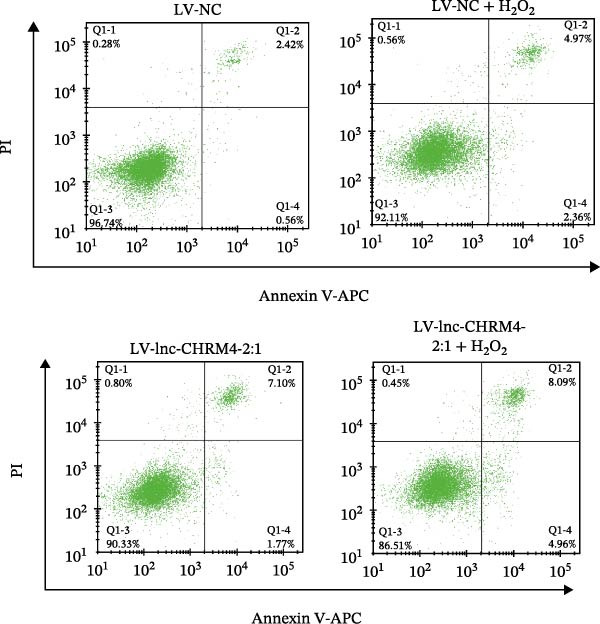
(F)

**Figure:**
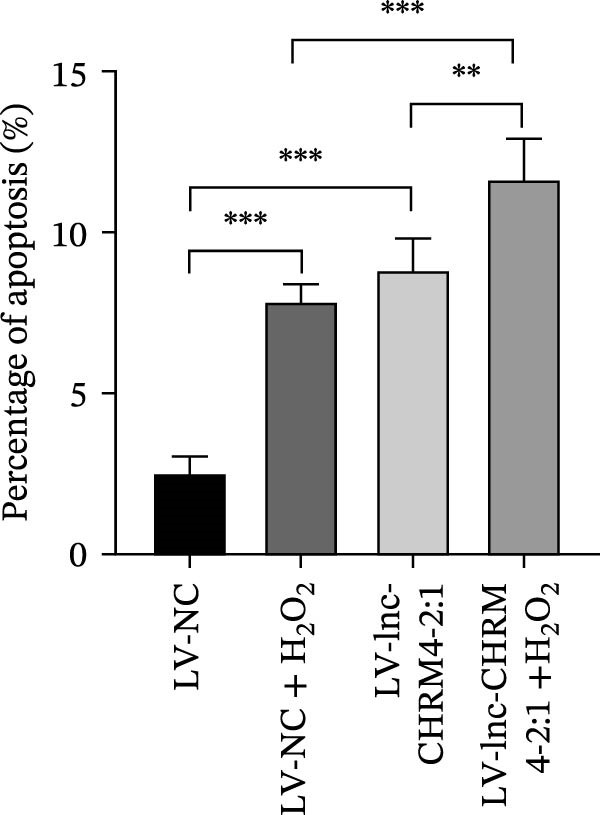
(G)

### 3.6. Lnc‐CHRM4‐2:1 Regulated the Proliferation and Apoptosis of Raw264.7 Cells

CCK‐8 results showed that compared with LV‐NC cells, days 0–3, lnc‐CHRM4‐2:1 had no significant effect on the proliferation of macrophages (Figure 6C). However, the cell proliferation activity increased significantly on the 4th day (*p* < 0.05) (Figure 6C), suggesting that lnc‐CHRM4‐2:1 could promote the proliferation of macrophages. EdU results suggested that overexpression of lnc‐CHRM4‐2:1 could promote the proliferation of Raw264.7 cells at 6 h, but the difference was not statistically significant (Figure 6D,E).

The percentage of apoptotic cells was detected by flow cytometry. We used 0.5 mM H_2_O_2_ to stimulate Raw264.7 cells overexpressed with lnc‐CHRM4‐2:1. Flow cytometry analysis showed that the proportion of apoptotic cells in lnc‐CHRM4‐2:1‐overexpressed Raw264.7 cells was significantly higher than that in the control group (8.85% ± 0.97% vs. 2.51% ± 0.55%, *p*  < 0.001). Similarly, the proportion of lnc‐CHRM4‐2:1‐overexpressed Raw264.7 cells stimulated by 0.5 mM H_2_O_2_ was also significantly higher than that in the control group stimulated by 0.5 mM H_2_O_2_ (11.67 ± 1.26% vs 7.86 ± 0.54%, *p* < 0.01) (Figure 6F,G) (Supporting Information 1). It could be concluded that overexpression of lnc‐CHRM4‐2:1 promoted the apoptosis of Raw264.7 cells.

### 3.7. Lnc‐CHRM4‐2:1 Regulated M1/M2 Polarization of Raw264.7 Cells

In order to explore the modifying effect of lnc‐CHRM4‐2:1 on M1/M2 macrophage polarization, LV‐NC and LV‐lnc‐CHRM4‐2:1 cells were treated with 100 ng/mL LPS, 20 ng/mL IFN‐γ, and 20 ng/mL IL‐4, respectively. After 6 h of exposure, the relative expressions of M1 macrophage markers (IL‐6, TNF‐α, and iNOS) and M2 macrophage markers (CD206, IL‐10, and Arg1) at the mRNA level were detected by qRT‐PCR. After 12 h of exposure, the protein expressions of iNOS and Arg1 in cells were detected by western blot. The results suggested that the expressions of IL‐6, TNF‐α, iNOS, CD206, IL‐10, and Arg1 at the mRNA level in LV‐lnc‐CHRM4‐2:1 group were not different from the LV‐NC group (all *p*  > 0.05) (Figure 7A–F). In contrast, the expression of Arg1 protein was decreased significantly in LV‐lnc‐CHRM4‐2:1 cells (*p* < 0.05) (Figure 7G,I) (Supporting Information 2). Besides, the expression of iNOS between the two groups at the protein level had no significant difference (*p* > 0.05) (Figure 7G,H) (Supporting Information 2). The expressions of IL‐6 and iNOS mRNAs in LV‐lnc‐CHRM4‐2:1 cells stimulated by LPS/IFN‐γ were significantly higher than that in LPS/IFN‐γ‐stimulated LV‐NC cells (all *p*  < 0.05) (Figure 7A,C), whereas there was rarely a significant difference in the expression of TNF‐α mRNA (*p*>0.05) (Figure 7B). Moreover, the expression of iNOS protein in LPS/IFN‐γ‐stimulated LV‐lnc‐CHRM4‐2:1 cells was significantly higher than that in LPS/IFN‐γ‐stimulated LV‐NC cells (*p* < 0.05) (Figure 7G,H), which was consistent with the result of the mRNA level (Figure 7C) (Supporting Information 2). Compared with IL‐4‐stimulated LV‐NC, the expression of Arg1 mRNA decreased significantly in LV‐lnc‐CHRM4‐2:1 cells stimulated by IL‐4 (*p*<0.01) (Figure 7F). However, there was no significant difference in the mRNA expressions of CD206 and IL‐10 (Figure 7D,E), and the protein expression of Arg1 (all *p* > 0.05) (Figure 7G,I) (Supporting Information 2). Taken together, the above results implicated that overexpression of lnc‐CHRM4‐2:1 could promote macrophage polarization into M1 cells.

Figure 7Effect of lnc‐CHRM4‐2:1 on M1/M2 polarization. The mRNA expression levels of M1 macrophage markers IL‐6 (A), TNF‐α (B), and iNOS (C) and M2 macrophage markers CD206 (D), IL‐10 (E), and Arg1 (F) were detected by qRT‐PCR. Western blot was used to detect the protein expression levels of iNOS and Arg1 in the cells of each group (G–I). Data were expressed as the mean ± SD, and were compared by ordinary one‐way ANOVA (*n* = 3,  ^∗∗∗∗^: *p* < 0.0001;  ^∗∗∗^: *p* < 0.001;  ^∗∗^: *p* < 0.01;  ^∗^: *p* < 0.05).

**Figure:**
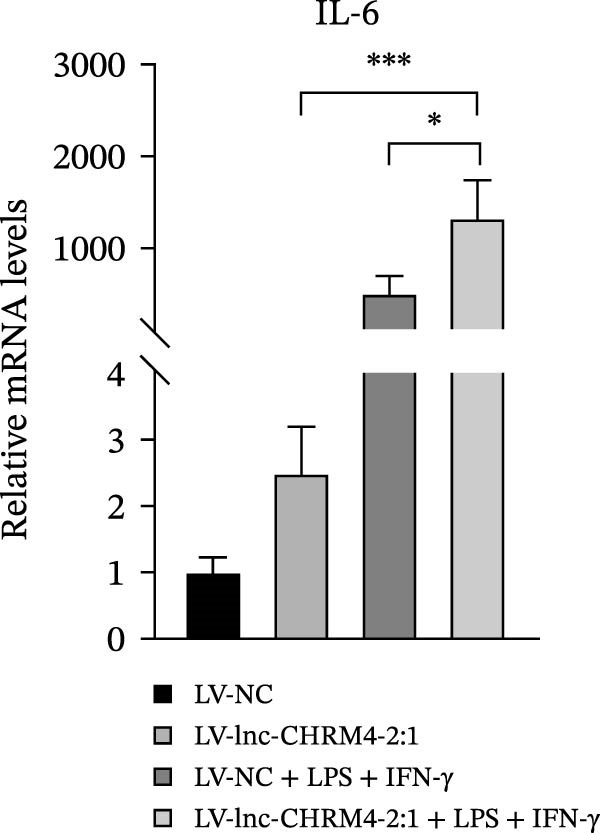
(A)

**Figure:**
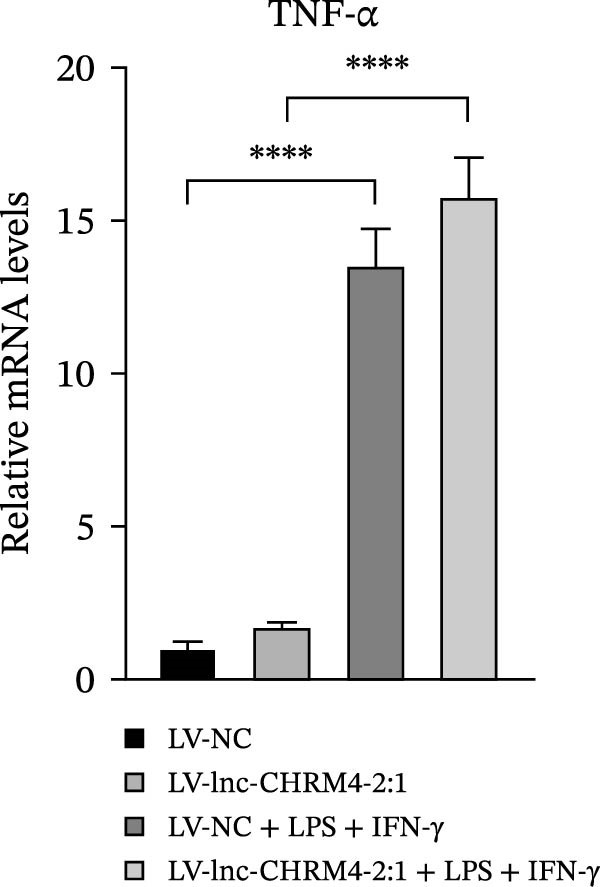
(B)

**Figure:**
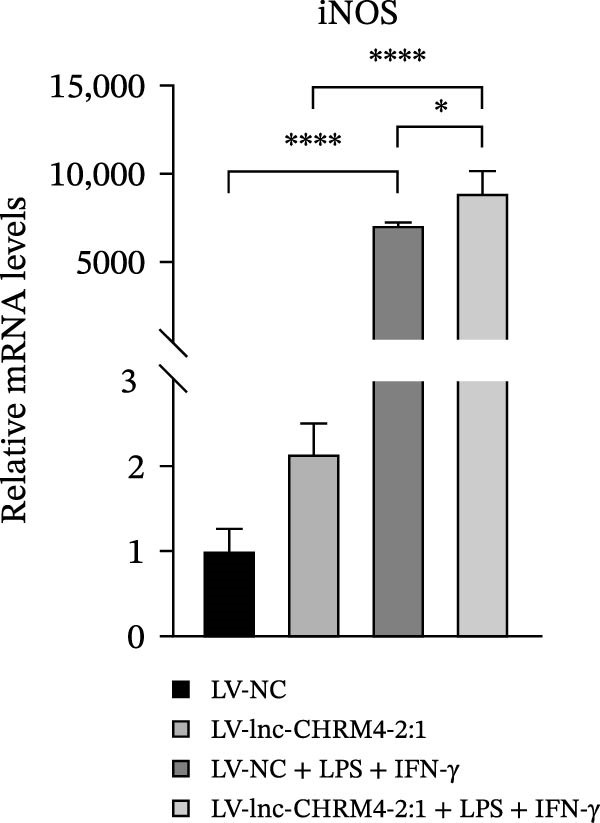
(C)

**Figure:**
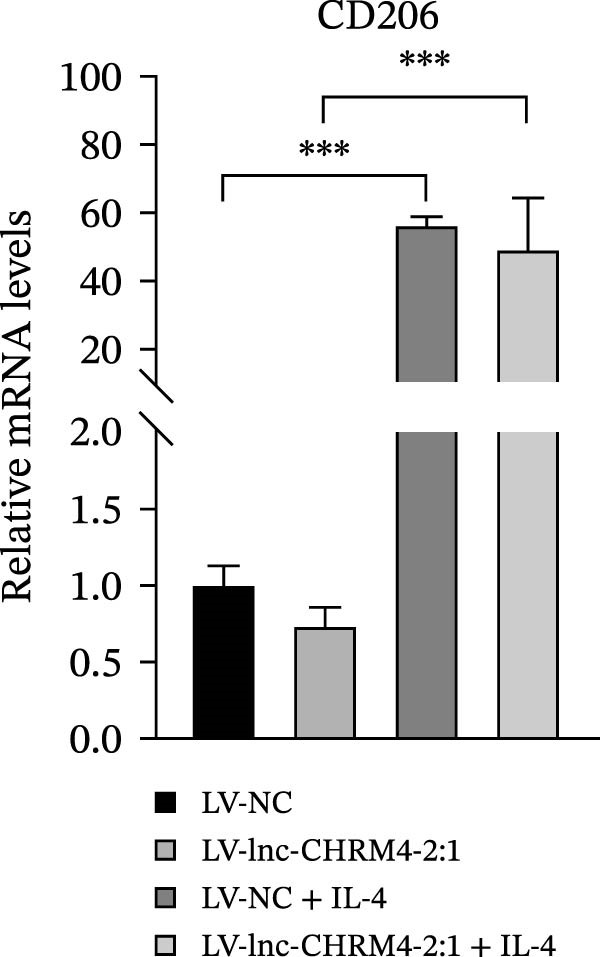
(D)

**Figure:**
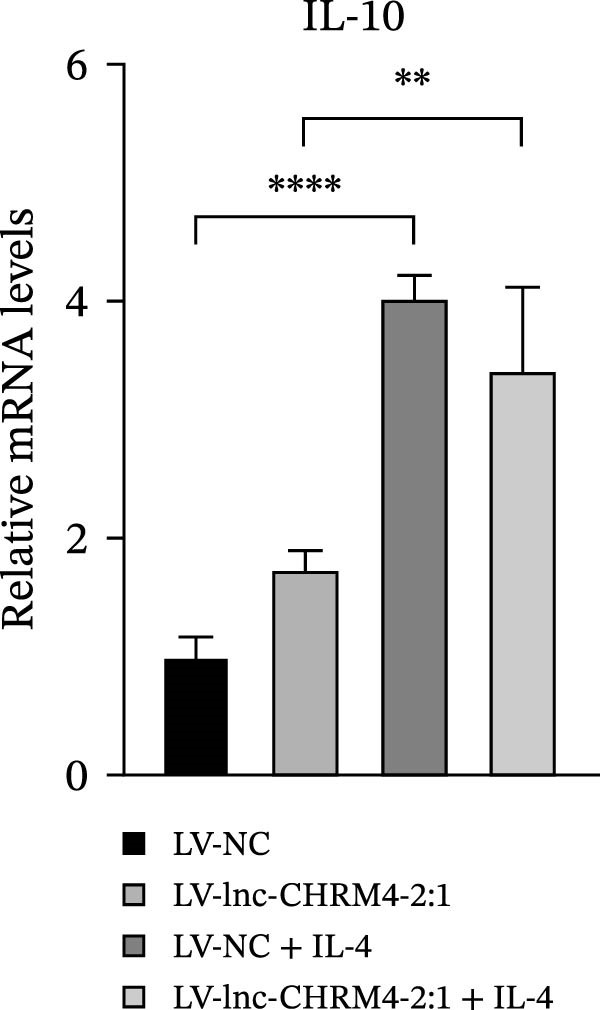
(E)

**Figure:**
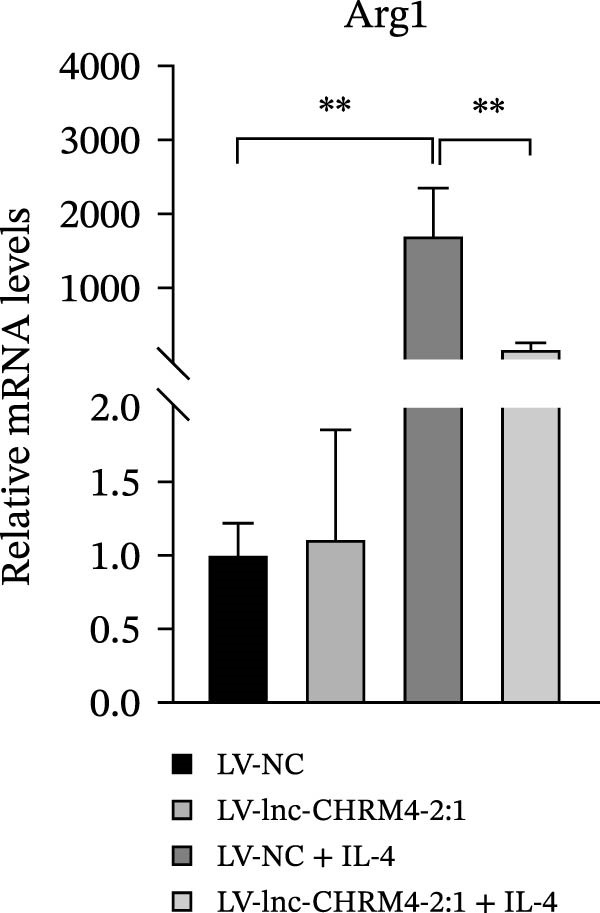
(F)

**Figure:**
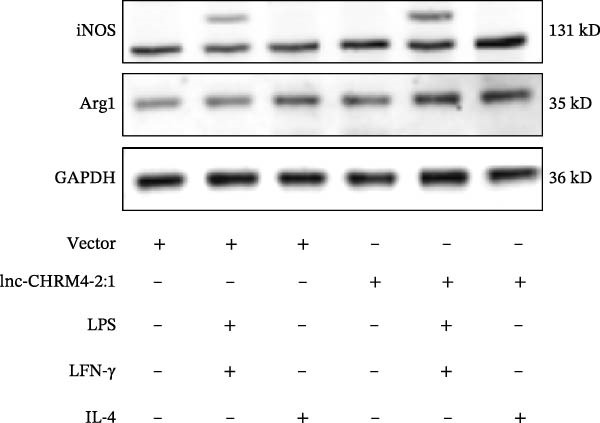
(G)

**Figure:**
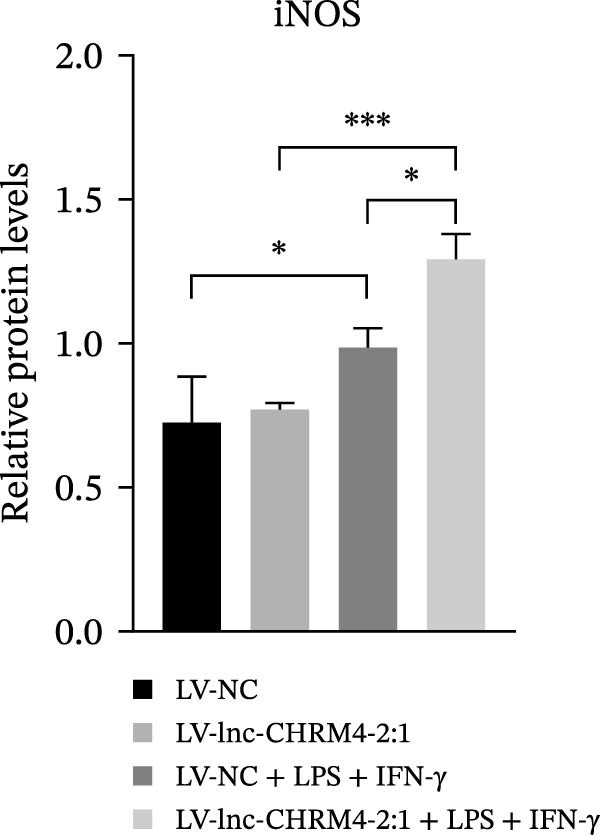
(H)

**Figure:**
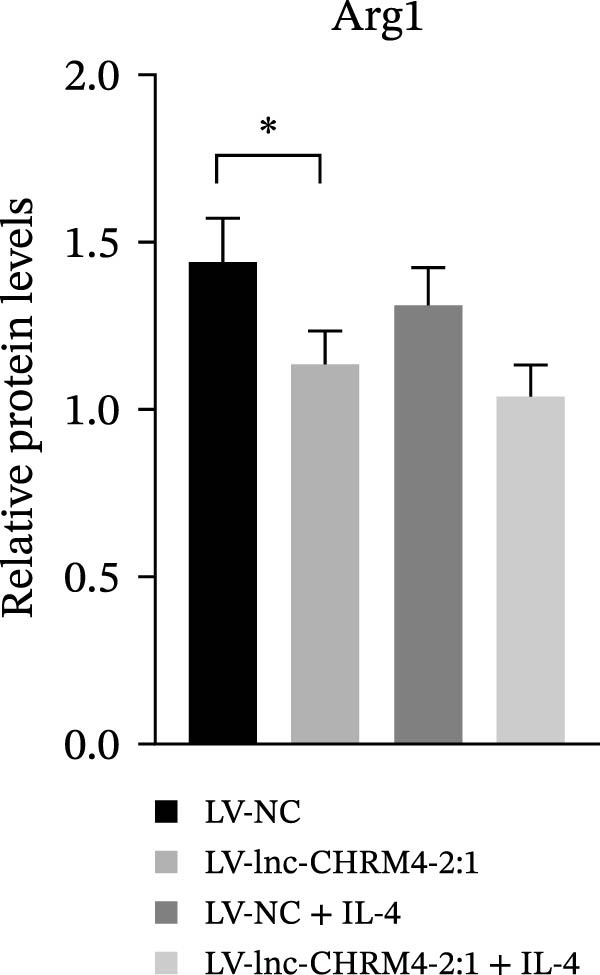
(I)

### 3.8. Lnc‐CHRM4‐2:1 Suppressed Macrophage Efferocytosis

The mRNA expression levels of MerTK, SLC2A1, SLC16A1, SLC7A11, MAF, GDF15, and MafB in LV‐NC and LV‐lnc‐CHRM4‐2:1 cells were detected by qRT‐PCR. The protein expression levels of MerTK and SLC2A1 were detected by western blot. The results showed that the mRNA expression levels of MerTK, SLC2A1, SLC16A1, and MafB in LV‐lnc‐CHRM4‐2:1 cells were significantly downregulated compared to those in LV‐NC cells (*p*<0.01, *p*  < 0.05, *p*  < 0.05, *p*  < 0.01) (Figure 8A–C,G), while SLC7A11 and MAF were significantly upregulated (*p*<0.05, *p*  < 0.001) (Figure 8D,E). There was no significant difference in the mRNA expression of GDF (Figure 8F). At the protein expression levels, we also found that the expression levels of MerTK and SLC2A1 in LV‐lnc‐CHRM4‐2:1 cells were significantly down‐regulated (all *p* < 0.05) (Figure 8H–J) (Supporting Information 2). To validate our findings, we performed an efferocytosis assay. Apoptosis was induced in Jurkat T cells via UV irradiation for 0–30 min. Flow cytometric analysis confirmed a significantly higher apoptosis rate after 20 min of irradiation (91.76 ± 3.02%) compared to the control (7.88 ± 1.49%) and 10‐min (53.00 ± 2.70%) groups (both *p* < 0.0001), while the 30‐min group showed no significant further increase (Figure 8K,L) (Supporting Information 1). Subsequently, Raw264.7 cells overexpressing lnc‐CHRM4‐2:1 or the corresponding control were cocultured with PKH26‐labeled apoptotic Jurkat T cells from the 20‐min irradiation group. Overexpression of lnc‐CHRM4‐2:1 significantly impaired the efferocytic capacity of Raw264.7 cells, as evidenced by a lower efferocytosis rate (14.97 ± 0.48%) compared to the control group (19.14 ± 2.11%; *p* < 0.05) (Figure 8M,N) (Supporting Information 1). These results suggested that lnc‐CHRM4‐2:1 significantly inhibited the efferocytosis of macrophages, which might be achieved by regulating the key functional molecules of MerTK and SLC2A1.

Figure 8The effect of lnc‐CHRM4‐2:1 on the efferocytosis of Raw264.7 cells. The mRNA expression levels of MerTK (A), SLC2A1 (B), SLC16A1 (C), SLC7A11 (D), MAF (E), GDF15 (F), and MafB (G) were detected by qRT‐PCR. Western blot was used to detect the protein expression levels of MerTK (H, I) and SLC2A1 (H, J) in the cells of each group. Data were expressed as the mean ± SD (*n* = 3). In (A, B, C, D, E, G, I, and J) data were analyzed using an unpaired *t*‐test; data in (F) were compared by the Mann–Whitney test ( ^∗∗∗^: *p* < 0.001;  ^∗∗^: *p* < 0.01;  ^∗^: *p* < 0.05; ns: not significant). Apoptosis of Jurkat T cells and macrophage efferocytosis were quantified by flow cytometry (K–N). The apoptosis rate was defined as the sum of early and late apoptotic populations. The efferocytosis rate was defined as the percentage of PKH26‐positive/Hoechst‐positive cells among all the Hoechst‐positive macrophages. Data were expressed as the mean ± SD. (L) Data were compared by ordinary one‐way ANOVA; data in (N) were compared by an unpaired *t*‐test (*n* = 3,  ^∗∗∗∗^: *p* < 0.0001; ^∗^: *p* < 0.05).

**Figure:**
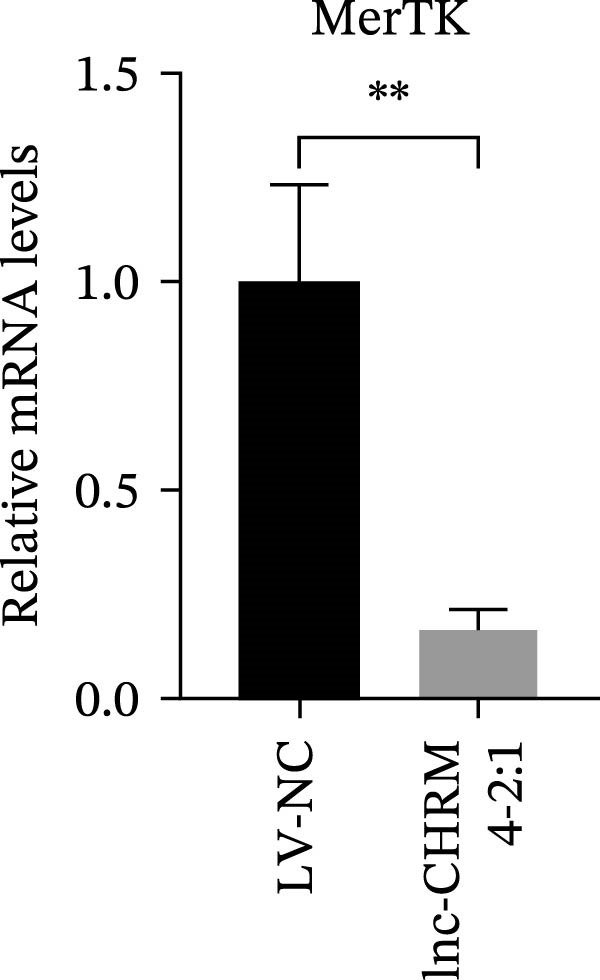
(A)

**Figure:**
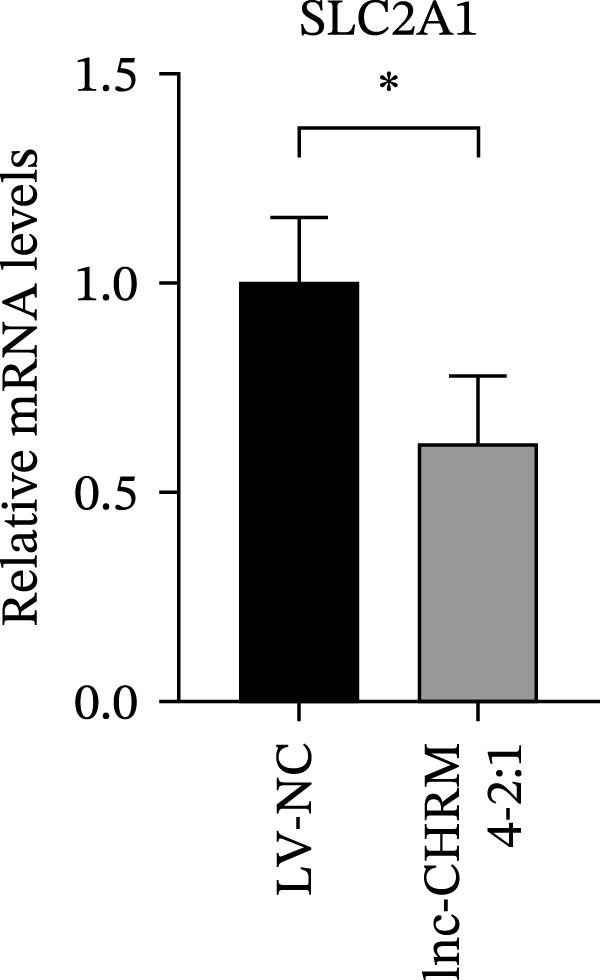
(B)

**Figure:**
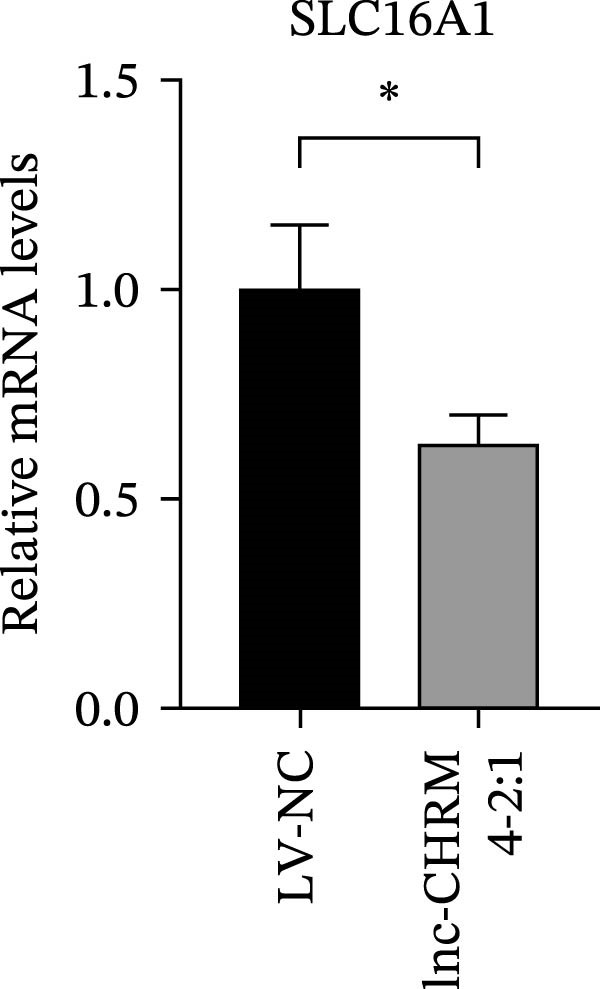
(C)

**Figure:**
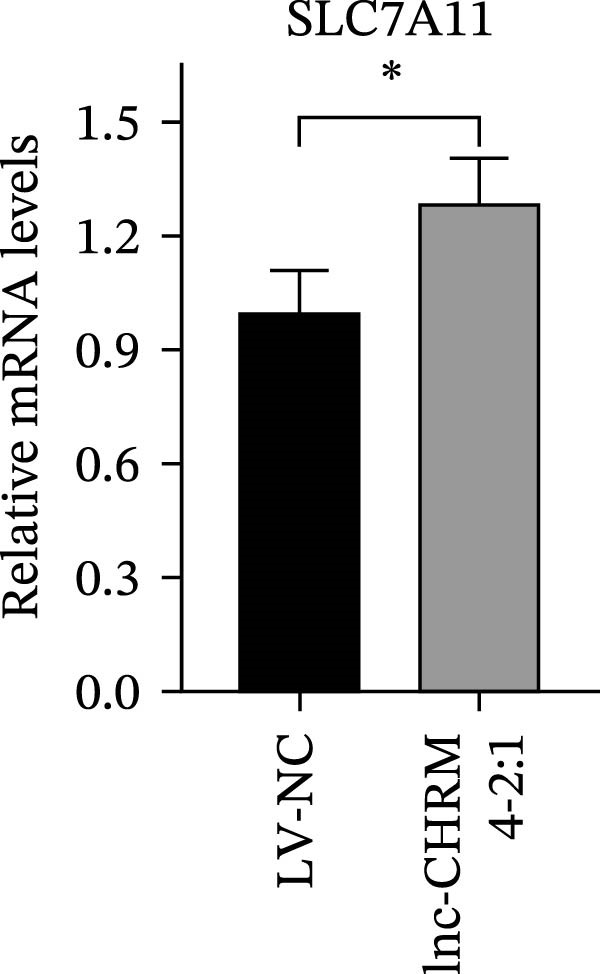
(D)

**Figure:**
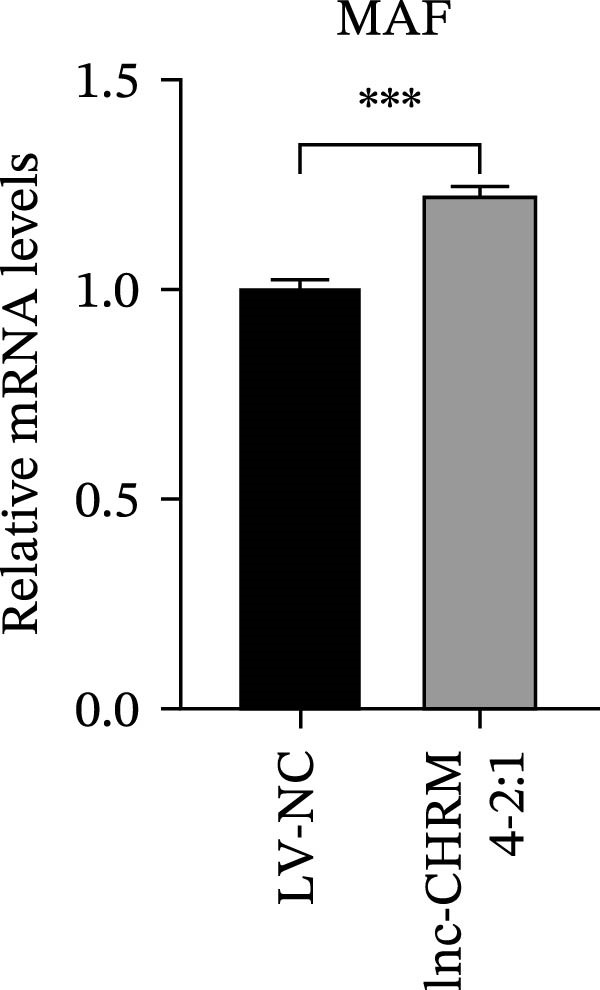
(E)

**Figure:**
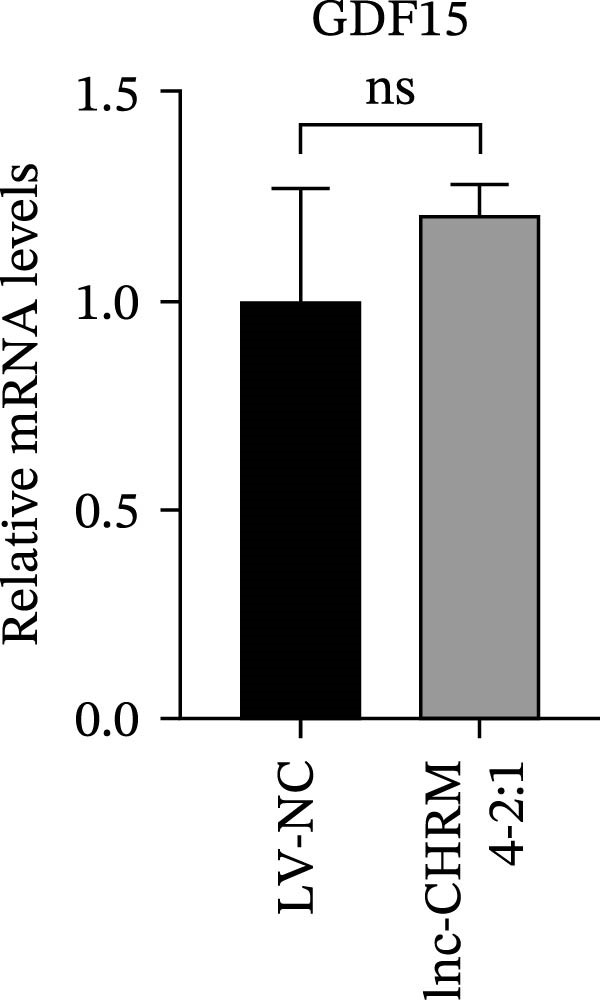
(F)

**Figure:**
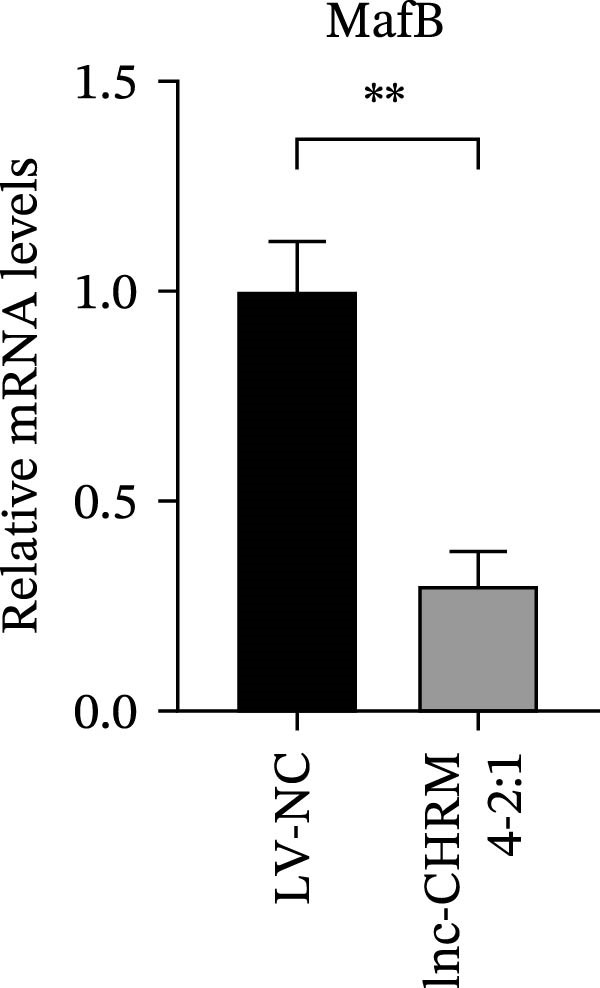
(G)

**Figure:**
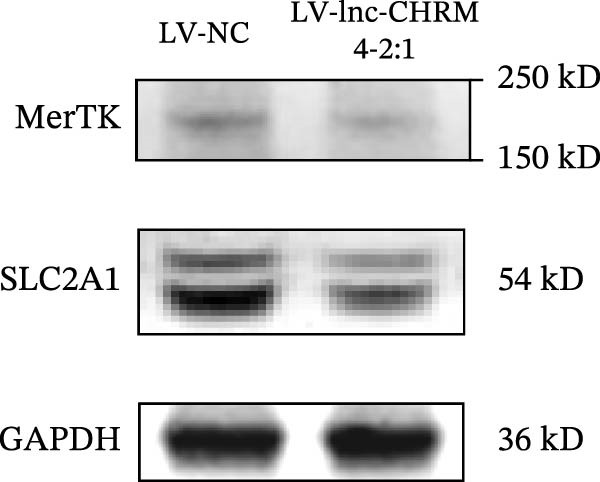
(H)

**Figure:**
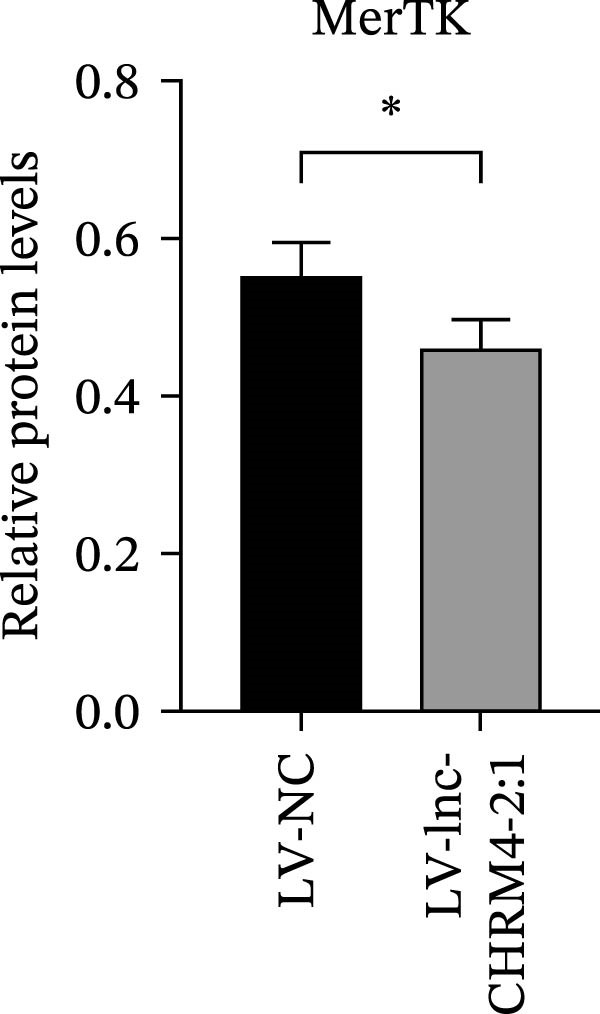
(I)

**Figure:**
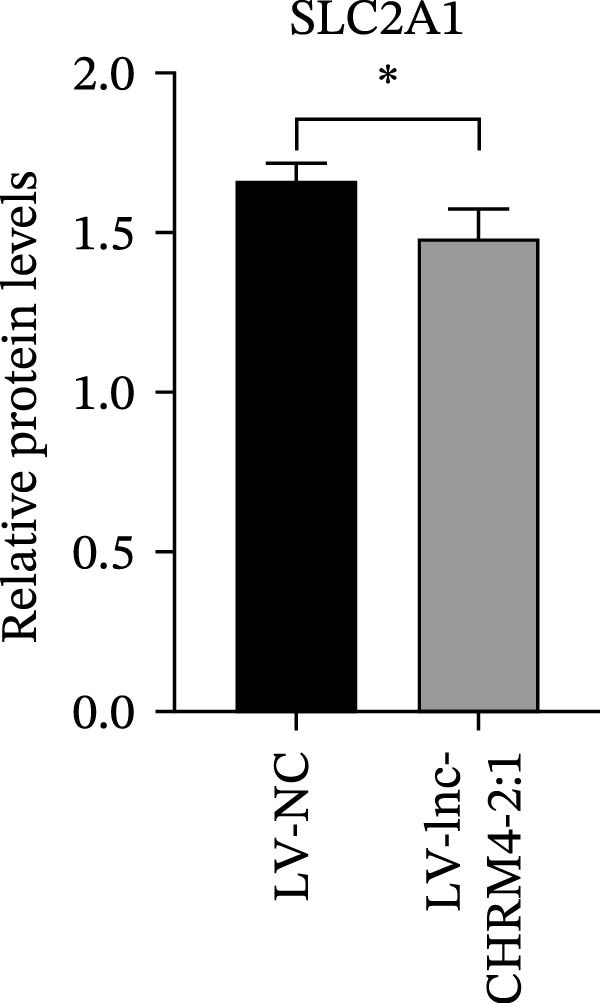
(J)

**Figure:**
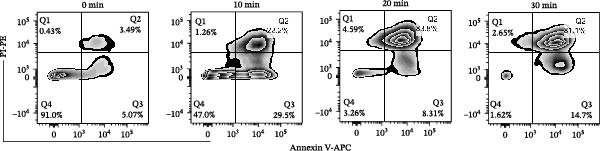
(K)

**Figure:**
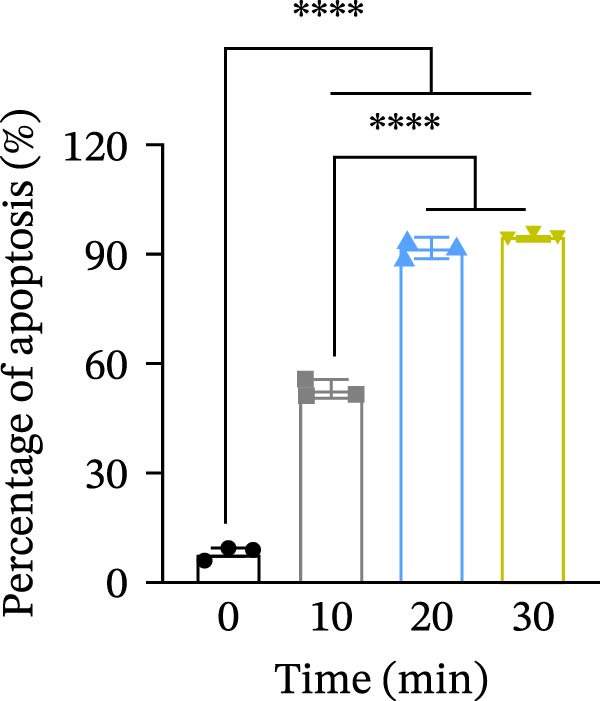
(L)

**Figure:**
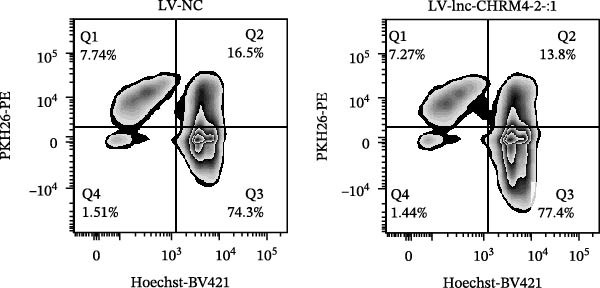
(M)

**Figure:**
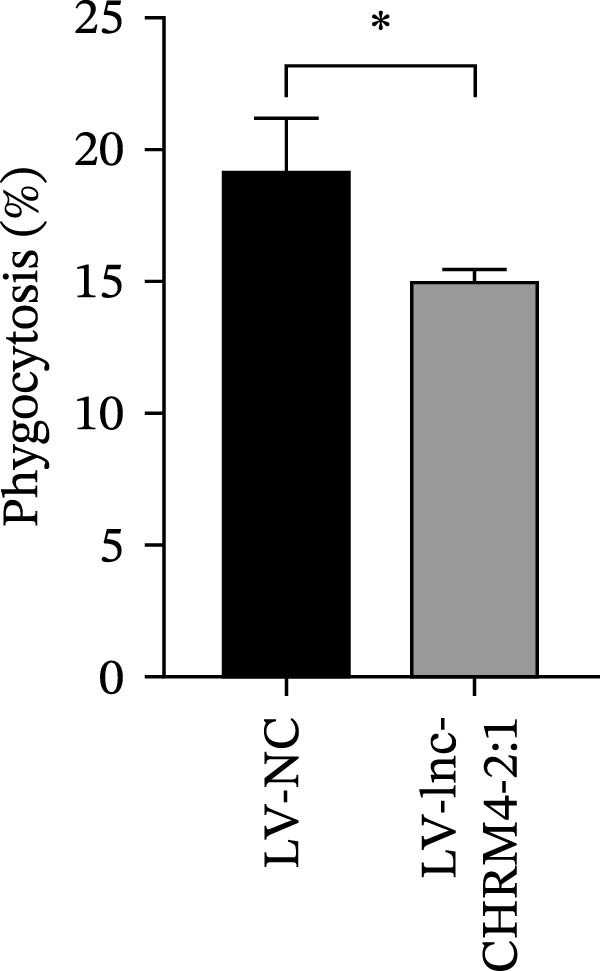
(N)

## 4. Discussion

RA is an autoimmune disease that mainly affects the peripheral joints and eventually leads to disability and loss of labor. It seriously endangers the physical and mental health of the patients and brings a great burden to society and families. The pathogenesis of RA has not been fully elucidated. Early diagnosis of RA is of great significance to improve the prognosis and quality of life of patients. Increasing evidence has shown that lncRNAs participate in the regulation of T cell response, macrophage response, synovial fibroblast proliferation, migration, invasion, autophagy, cartilage injury and repair, and the balance of immune microenvironment in joint region through RNA–RNA and RNA–protein interaction in RA [15, 25–30]. Therefore, identification of RA‐specific lncRNAs may provide insight into the exploration of novel strategies for the diagnosis and treatment of RA.

Luo et al. [31] have explored the disease‐specific lncRNA expression profile of RA for the first time using a human lncRNA microarray gene chip. The lncRNA expression profile of PBMCs in middle‐aged female patients with RA‐associated interstitial lung disease (RA‐ILD) was analyzed, and four specific biomarkers were screened for RA, including NR_002819 (MALAT1), NR_038935, ENST00000603415, and ENST00000560199, confirming the important clinical significance of lncRNA as a specific biomarker of RA in disease diagnosis [32]. The expression of lncRNAs in fibroblast‐like synoviocytes (RA‐FLS) in RA was screened by lncRNA microarray, suggesting that lncRNA was closely related to RA‐FLS autophagy [33]. ceRNA is a key mechanism for lncRNA. Salmena et al. [34] proposed the hypothesis of ceRNA in 2011, that is, transcripts such as lncRNA, circRNA, and mRNA competitively bind to miRNA through miRNA binding sites to form a ceRNA regulatory network. A previous study has found that lncRNA PICSAR might act as a ceRNA molecule to antagonize the effect of miR‐4701‐5p in RA, thereby aggravating synovial invasion and joint destruction [35]. Hence, the identification of RA‐specific lncRNA expression profile and the functional network in specific immune cell subsets is of great importance to clarify the pathogenesis of RA and explore promising diagnostic markers and potential immunotherapeutic targets. In our previous study, we found that lncRNA HIX003209, which was dysregulated in RA, could sponge miR‐6089 to inhibit the posttranscriptional regulation of miR‐6089 on the targeted gene Toll‐like receptor 4 (TLR4), thereby promoting TLR4‐mediated macrophage response in RA [28]. In this study, through high‐throughput sequencing and qRT‐PCR mRNA expression verification, we found the dysregulated lncRNAs in PBMCs of RA patients, including significantly upregulated lncRNAs of lnc‐CHRM4‐2:1, lnc‐SLFN5‐1:4, lnc‐NBPF19‐5:1, and lnc‐ZNF253‐2:7, and downregulated lncRNAs of lnc‐BCL2A1‐3:2, TCONS_00027971, lnc‐IL1B‐2:1, lnc‐ARHGAP29‐7:1. Among them, lnc‐CHRM4‐2:1 was the most differentially expressed lncRNA in RA. Further study implicated that there was no significant correlation between the expression of lnc‐CHRM4‐2:1 and the clinical indexes of RA, such as PLT, RF, CRP, and ESR, besides CCP, indicating an indefinite relationship with general disease activity but a potential connection to CCP‐specific autoimmune pathogenesis and disease subtypes. Nonetheless, future large‐scale, prospective studies are warranted to validate its utility in stratifying patient populations and to elucidate the functional role of lnc‐CHRM4‐2:1 and its power as a diagnostic marker in RA.

Macrophages are one of the key immune cells mediating the innate immune response. Changes in their metabolic status and immune function affect the body’s immune balance and inflammatory response. Macrophage extracellular DNA traps (METs) are reported to promote the proliferation and migration of RA‐FLS, which mediates inflammatory response by releasing DNA to extracellular space and activating cyclic GMP‐AMP synthase (cGAS) in RA [36, 37]. Macrophage inflammatory cascade‐related cytokines and enzymes can activate osteoclasts, and fibroblast synovial cells play key roles in the occurrence and development of RA, leading to joint destruction and disease progression [36, 37]. Under the state of inflammation and immune activation, macrophages can polarize into proinflammatory M1 phenotype, producing a large number of proinflammatory factors, such as TNF‐α, IL‐6, IL‐1β, and iNOS. M1 macrophages aggravate the inflammatory response of cartilage and synovium, induce osteoclast proliferation and differentiation and osteoblast apoptosis, thereby aggravating bone erosion and joint damage [38, 39]. On the contrary, M2 macrophages mainly generate anti‐inflammatory factors, such as IL‐10, TGF‐β, and Arg1, which exert anti‐inflammatory effects and help to repair the injured tissues by promoting angiogenesis and reducing cartilage injury [40]. Therefore, the maintenance of M1/M2 polarization balance is essential for controlling the development and progression of RA. Recent studies have demonstrated that multiple lncRNAs modulate macrophage polarization and are intimately associated with RA pathogenesis. Specifically, lncRNA H19, lncRNA‐ANRIL, and MALAT1 are markedly upregulated in RA patients and function as pivotal regulators of M1 macrophage polarization, thereby enhancing macrophage‐mediated inflammatory responses and accelerating disease progression [15–17]. In the current study, we have demonstrated that the upregulation of lnc‐CHRM4‐2 :1 expression could promote Raw264.7 macrophage proliferation and M1 polarization, suggesting a pathogenic role of lnc‐CHRM4‐2:1 in RA by regulating macrophage polarization. Moreover, currently published studies have suggested a variety of key molecules involved in regulating RA by affecting macrophages differentiation, polarization, efferocytosis, and biological functions, such as MafB, MAF, SLC16A1, SLC7A11, SLC2A1, and GDF‐15. MafB is a member of the MAF transcription factor family. MafB plays an anti‐inflammatory role in RA by promoting the activation of M2 macrophages [41]. MAF is another important member of the large MAF family, which is involved in RA pathogenesis [42]. Both MafB and MAF are essential factors regulating the biological function of macrophages. SLC16A1 promotes M2 polarization by regulating lactate uptake of macrophages in RA [43]. SLC7A11 plays an important regulatory role in RA by regulating FLS cell proliferation and migration [44]. SLC2A1, also known as glucose transporter 1 (GLUT1), participates in the regulation of metabolic reprogramming of the joint in RA [45, 46]. GDF‐15 is a member of the transforming growth factor‐β (TGF‐β) superfamily. It has been documented that plasma GDF‐15 is elevated in patients with RA [47]. Besides, the gene polymorphism of GDF‐15 is associated with the risk of RA in the Chinese Han population [47]. The above findings have all implicated that MafB, MAF, SLC16A1, SLC2A1, and GDF15 are critical regulators in RA by influencing macrophage polarization, efferocytosis, and metabolic reprogramming, serving as promising markers for RA. In this study, we constructed a ceRNA network of lnc‐CHRM4‐2:1‐miRNA–mRNA based on miRanda database prediction. We speculate that lnc‐CHRM4‐2:1 might be involved in the regulation of macrophage polarization and efferocytosis and thereby contribute to RA pathogenesis by sponging miRNAs that target key genes such as MerTK, MafB, MAF, SLC16A1, SLC2A1, and GDF15.

Efferocytosis is the process of phagocytosis and clearance of apoptotic cells by macrophages and other phagocytic cells, which is crucial for maintaining homeostasis and tissue and organ integrity [18]. The impairment of efferocytosis is closely related to inflammatory and immune disorders [48, 49]. It has been reported that lncRNA MIAT positively regulates the expression of antiphagocytic molecule CD47 through sponging miR‐149‐5p, inhibiting the efferocytosis of macrophages and promoting the progress of atherosclerosis [50]. LncRNA SCARNA8 promoted atherosclerotic plaque instability by inhibiting macrophage efferocytosis through targeting PPAR [51]. The efferocytosis‐related lncRNAs identified from The Cancer Genome Atlas (TCGA) database demonstrated robust, independent prognostic value for patients with clear‐cell renal cell carcinoma (ccRCC) as well as pancreatic adenocarcinoma [52, 53]. MerTK, as a receptor on the surface of macrophages, can bind to the “eat me” signal phosphatidylserine (PS) exposed on the surface of apoptotic cells and promote the phagocytosis of apoptotic cells by macrophages. It has been well documented that MerTK‐mediated macrophage efferocytosis plays a protective role in defending against joint inflammation in an RA mouse model [4]. Macrophage‐specific lncRNA MAARS (macrophage‐associated atherosclerosis lncRNA sequence) has been proven to be a key regulator of macrophage apoptosis and efferocytosis in vitro [54]. LncRNA MAARS mediates macrophage efferocytosis by targeting MerTK and plays an important role in the progression of atherosclerotic plaque, suggesting the critical role of lncRNA in regulating macrophage efferocytosis by targeting MerTK [54]. However, the effect of lncRNA on macrophage efferocytosis in RA has not been reported. Lnc‐CHRM4‐2:1 is a well‐established RA‐specific lncRNA, possessing the potential to regulate macrophage differentiation and function by targeting MerTK in RA. In this study, mouse Raw264.7 overexpressing lnc‐CHRM4‐2:1 was constructed by lentivirus infection to estimate the role of lnc‐CHRM4‐2:1 in regulating macrophages in RA. In vitro experiments, upregulation of lnc‐CHRM4‐2:1 had been demonstrated to promote the proliferation, apoptosis, and M1 polarization of Raw264.7 macrophages but inhibit the efferocytosis and M2 polarization. Taken together, we conclude that lnc‐CHRM4‐2:1 plays a pathogenic role in RA by inhibiting anti‐inflammatory M2 macrophage polarization and efferocytosis, thus potentially promoting immune and inflammatory responses in RA.

Previous studies have shown that TLR4/NF‐κB signal pathway affects the progress of RA by promoting M1 polarization of macrophages [55]. Li et al. [56] have established a plant drug‐component‐center‐target‐disease network and confirmed that these traditional Chinese medicine components can inhibit immune and inflammatory responses, reduce chemokine release, and suppress the destruction of RA synovial extracellular matrix by regulating the IL‐17 signaling pathway, TNF signaling pathway, and TLR signaling pathway. In this study, differentially expressed lncRNAs in PBMCs of RA patients were screened by full transcriptome sequencing, and their biological functions and potential regulatory mechanisms were explored by GO functional enrichment and KEGG pathway analyses. It showed that these dysregulated lncRNAs in RA were involved in various organic acid metabolism and lipid biosynthesis, and some lncRNAs were involved in the formation of mitochondrial ribosomes, cortical endoplasmic reticulum, and other membrane components. Similar to previous findings, the result of KEGG pathway analysis had suggested that lncRNAs were mainly involved in the regulation of RA by regulating fatty acid biosynthesis/degradation, cytokine–cytokine receptor interaction, chemokine signaling pathway, IL‐17 signaling pathway, TLR signaling pathway, and NF‐κB signaling pathway. Therefore, dysregulated expression of lncRNAs may participate in the pathogenesis of RA by regulating immune and inflammatory responses through the classic signaling pathways, including IL‐17 signaling, TLR/NF‐κB signaling, and TNF signaling. More future studies are warranted for the mechanistic study of the pathogenesis of RA regarding the specifically dysregulated lncRNAs in RA.

There are some limitations in this study. First of all, it is necessary to increase the sample size and refine the grouping according to the disease activity score and other indicators. Second, the effects of lnc‐CHRM4‐2:1 on proliferation, apoptosis, efferocytosis, and M1/M2 polarization of macrophages remain to be further clarified using an lnc‐CHRM4‐2:1‐knockout cell model and in vivo study. Third, we recognize that the computationally predicted ceRNA network requires direct experimental validation. Consequently, we plan to perform RNA pull‐down and dual‐luciferase reporter assays as an immediate future priority to definitively establish the interactions between lnc‐CHRM4‐2:1, miRNAs, and target mRNAs. Fourth, the findings from our study, which exclusively employed the mouse Raw264.7 macrophage model, warrant further validation in human macrophages (e.g., the THP‐1 cell line), given the well‐documented interspecies differences in origin, metabolism, inflammatory response, and phagocytic function [53, 56]. Last but not least, currently available data have strongly supported the critical role of METs in mediating immune and inflammatory responses in RA. We hypothesize that METs might be involved in the regulation of lnc‐CHRM4‐2:1‐mediated biological functions in RA through the DNA–RNA interaction. Therefore, the biological function and mechanism of lnc‐CHRM4‐2:1 should be further verified by animal experiments, which will provide novel insight into the exploration of new strategies for the diagnosis and treatment of RA.

In summary, this study suggests that lnc‐CHRM4‐2:1 is specifically dysregulated in RA. Lnc‐CHRM4‐2:1 can promote macrophage proliferation and M1 polarization but inhibit cell efferocytosis and M2 polarization in RA, playing an important role in the pathogenesis of RA by regulating the polarization and efferocytosis of macrophages. Lnc‐CHRM4‐2:1 is expected to be a potential RA‐specific biomarker and a new therapeutic target for RA.

## Author Contributions

Jinjin Chu and Donghua Xu designed the experiments. Jinjin Chu and Lili Zhang carried out the experiments. Zhuojian Qu, Haibo Li, and Chunjuan Yang collected the data. Jiamei Sun and Linlin Gai carried out the data analyses. Jinjin Chu, Jie Zang, and Donghua Xu wrote and revised the article. Xuecheng Sun helped to revise the article.

## Funding

This study was supported by the National Natural Science Foundation of China (Grants 82171790 and 82402112), the Science and Technology Development Program of Weifang (Grant 2022YX079), the Weifang Municipal Health Commission Research Project (Grant WFWSJK‐2024‐003), the Natural Science Foundation of Shandong Province (Grants ZR2024MH079, ZR2024QH228, and ZR2024QC071), the Science and Technology Development Plan of Shandong Province (Grant 202401060357), and the Shandong Medical Health Science and Technology project (Grant 202403110363).

## Disclosure

All authors have read and approved the final submitted manuscript.

## Ethics Statement

This study was approved by the Ethics Committee of Weifang People’s Hospital, Shandong Second Medical University (Number 2020YX009).

## Consent

All patients provided informed consent.

## Conflicts of Interest

The authors declare no conflicts of interest.

## Supporting Information

Additional supporting information can be found online in the Supporting Information section.

## Supporting information


**Supporting Information 1** Raw Data_Flow Cytometry.zip: original flow cytometry data of all samples.

**Supporting Information 2** Figure 7G, Figure 8H .Raw Data_Western Blots.docx: original Western blot data and experimental parameters. All raw data match the experimental results in the main manuscript and support result validation.

## Data Availability

All sequencing data were deposited in the Genome Sequence Archive for Human (GSA‐Human) database under Accession Number HRA007699.
