# Supplementary material for: Lnc‐CHRM4‐2:1 Inhibits M2 Polarization and Efferocytosis of Macrophages by Downregulating MerTK and SLC2A1 in Rheumatoid Arthritis
Source: J Immunol Res. 2026 Feb 27;2026:1718207. doi: 10.1155/jimr/1718207 (PMC13140872; doi:10.1155/jimr/1718207)
Supplement: Supplementary file 1 — Supporting Information 1 Raw Data_Flow Cytometry.zip: original flow cytometry data of all samples. [file JIMR-2026-1718207-s002.zip › Supplementary Raw Data_Flow Cytometry/Fig. 6F 6G/LV-lnc-CHRM4-2 1/2.pdf]

流式细胞术检测报告单

姓名： 年龄： 性别：
病历号： 科室： 床号：
主管医生： 样本类型： 采样时间：

0-1[参数]	全称	简称	结果	单位	指示	参考范围

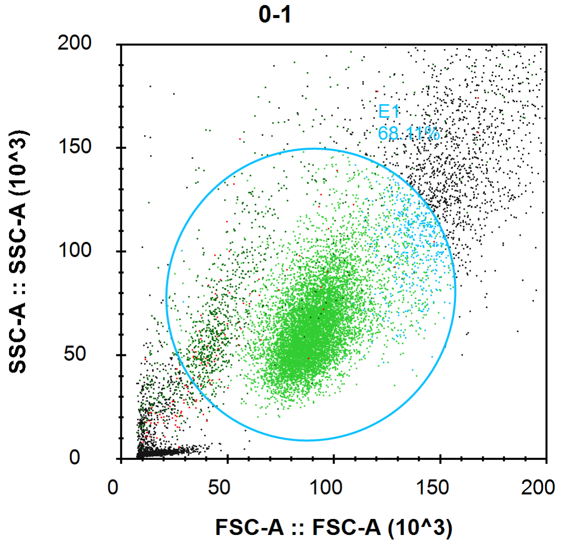

Gate	Count	%All	Mean X	Median X
All	14724	100.00%	26750	89180
E1	10029	68.11%	89906	89331

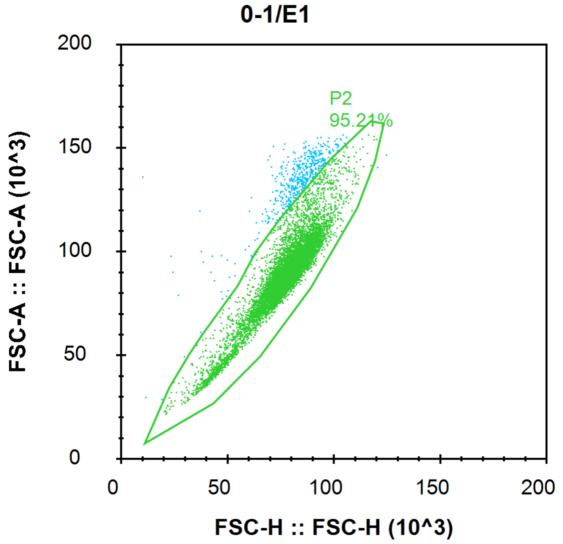

Gate	Count	%E1	Mean X	Median X
E1	10029	100.00%	76778	78425
P2	9549	95.21%	76492	78130

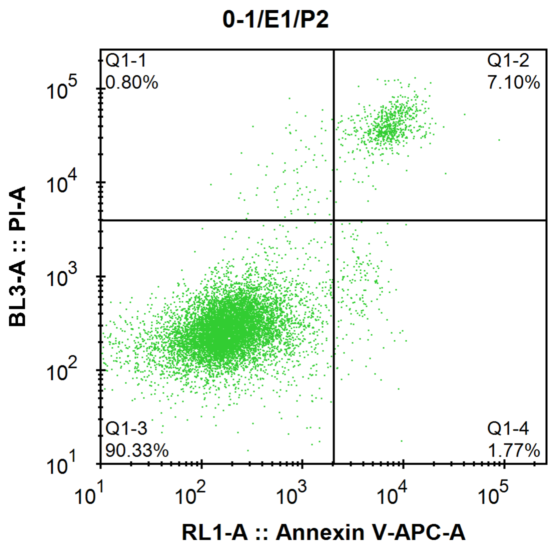

Gate	Count	%P2	Mean X	Median X
P2	9549	100.00%	874	193
Q1-1	76	0.80%	947	887
Q1-2	678	7.10%	8050	7361
Q1-3	8626	90.33%	242	178
Q1-4	169	1.77%	4349	3776

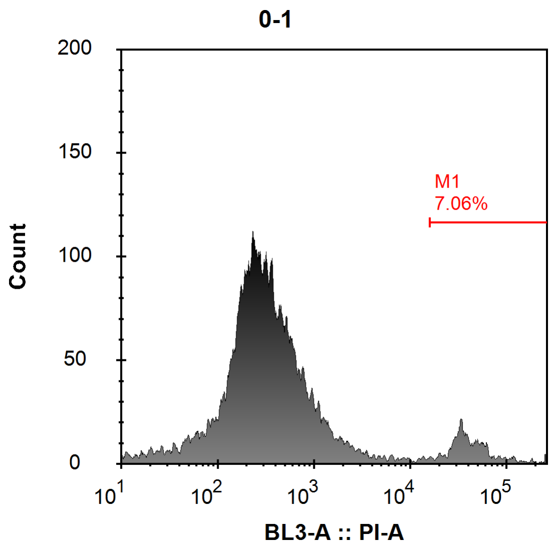

Gate	Count	%All	Mean X	Median X
All	14724	100.00%	4424	301
M1	1040	7.06%	55324	39477

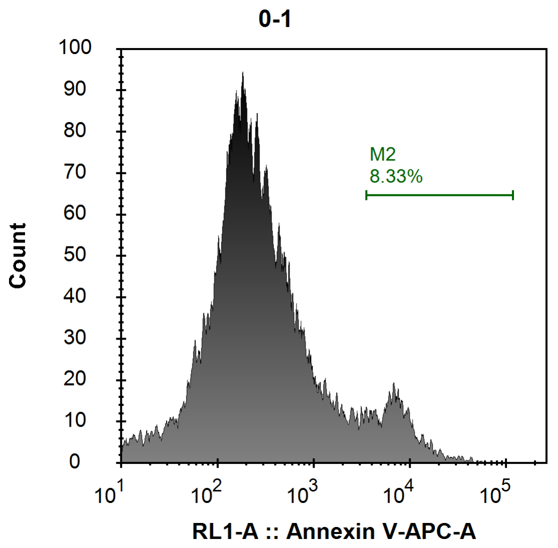

Gate	Count	%All	Mean X	Median X
All	14724	100.00%	1174	219
M2	1227	8.33%	9479	7227
