# Supplementary material for: Lnc‐CHRM4‐2:1 Inhibits M2 Polarization and Efferocytosis of Macrophages by Downregulating MerTK and SLC2A1 in Rheumatoid Arthritis
Source: J Immunol Res. 2026 Feb 27;2026:1718207. doi: 10.1155/jimr/1718207 (PMC13140872; doi:10.1155/jimr/1718207)
Supplement: Supplementary file 1 — Supporting Information 1 Raw Data_Flow Cytometry.zip: original flow cytometry data of all samples. [file JIMR-2026-1718207-s002.zip › Supplementary Raw Data_Flow Cytometry/Fig. 6F 6G/LV-lnc-CHRM4-2 1+H2O2/1.pdf]

流式细胞术检测报告单

姓名：	年龄：	性别：
病历号：	科室：	床号：
主管医生：	样本类型：	采样时间：

0.5-1[参数]	全称	简称	结果	单位	指示	参考范围

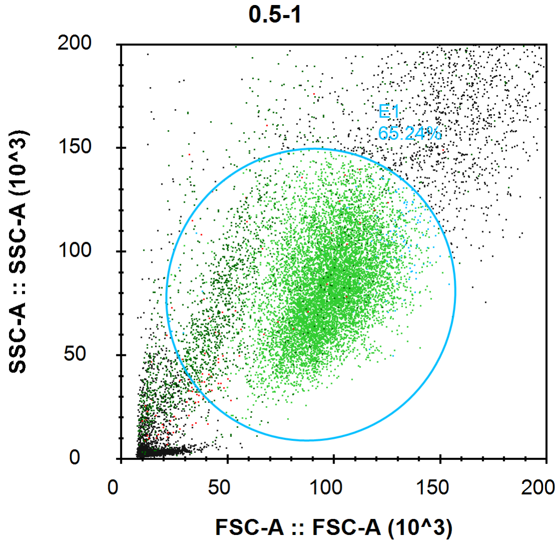

Gate	Count	%All	Mean X	Median X
All	15362	100.00%	31314	92764
E1	10022	65.24%	92718	95088

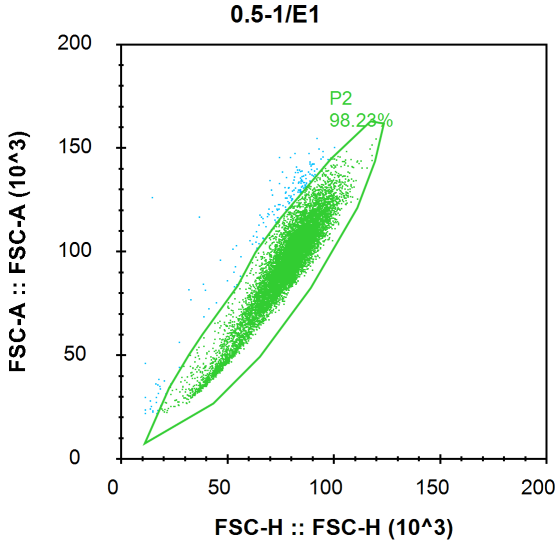

Gate	Count	%E1	Mean X	Median X
E1	10022	100.00%	76553	78833
P2	9845	98.23%	76717	78849

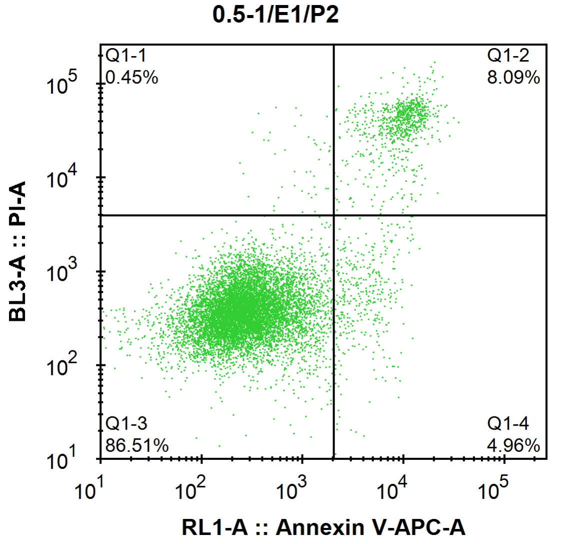

Gate	Count	%P2	Mean X	Median X
P2	9845	100.00%	1399	294
Q1-1	44	0.45%	1043	1055
Q1-2	796	8.09%	10271	10019
Q1-3	8517	86.51%	357	254
Q1-4	488	4.96%	5134	4331

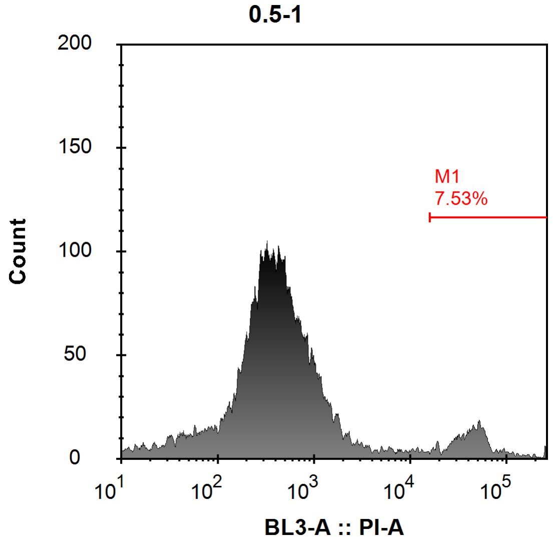

Gate	Count	%All	Mean X	Median X
All	15362	100.00%	5083	392
M1	1157	7.53%	58969	45651

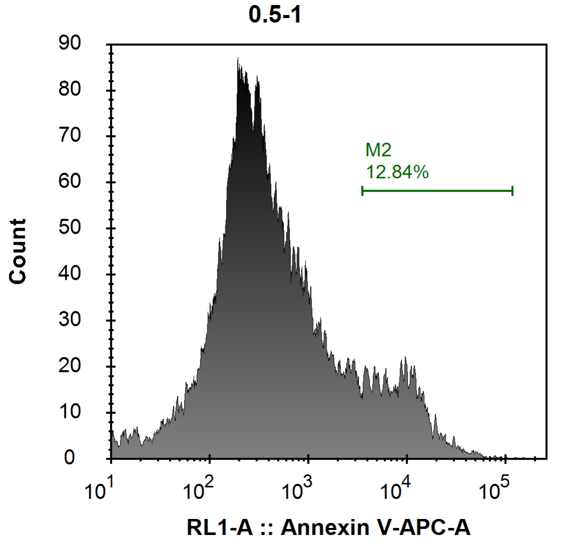

Gate	Count	%All	Mean X	Median X
All	15362	100.00%	1923	326
M2	1972	12.84%	11267	8721
