# Supplementary material for: Lnc‐CHRM4‐2:1 Inhibits M2 Polarization and Efferocytosis of Macrophages by Downregulating MerTK and SLC2A1 in Rheumatoid Arthritis
Source: J Immunol Res. 2026 Feb 27;2026:1718207. doi: 10.1155/jimr/1718207 (PMC13140872; doi:10.1155/jimr/1718207)
Supplement: Supplementary file 1 — Supporting Information 1 Raw Data_Flow Cytometry.zip: original flow cytometry data of all samples. [file JIMR-2026-1718207-s002.zip › Supplementary Raw Data_Flow Cytometry/Fig. 6F 6G/LV-NC/2.pdf]

流式细胞术检测报告单

姓名： 年龄： 性别：
病历号： 科室： 床号：
主管医生： 样本类型： 采样时间：

0[参数]	全称	简称	结果	单位	指示	参考范围

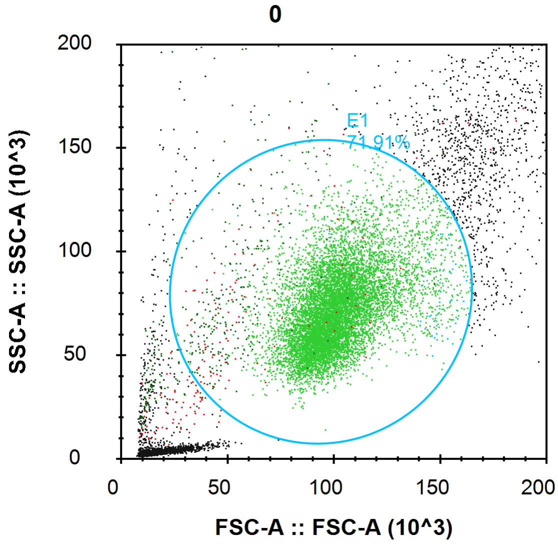

Gate	Count	%All	Mean X	Median X
All	13910	100.00%	97288	98044
E1	10003	71.91%	100517	99386

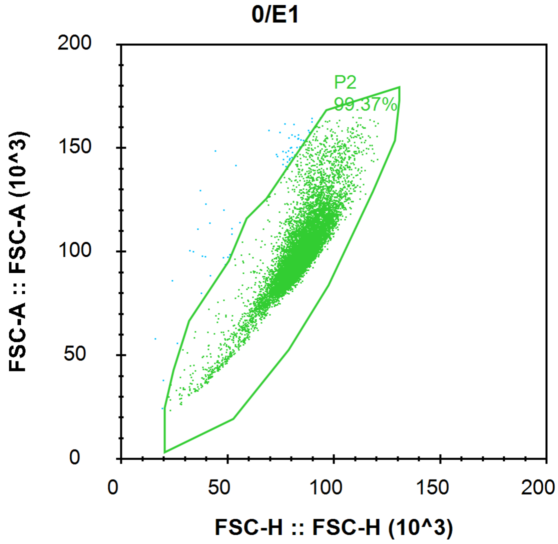

Gate	Count	%E1	Mean X	Median X
E1	10003	100.00%	84100	85234
P2	9940	99.37%	84224	85283

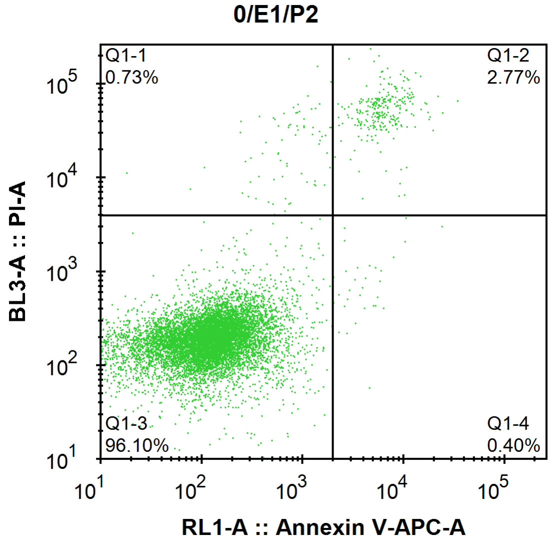

Gate	Count	%P2	Mean X	Median X
P2	9940	100.00%	377	123
Q1-1	73	0.73%	855	737
Q1-2	275	2.77%	7127	6222
Q1-3	9552	96.10%	160	119
Q1-4	40	0.40%	4836	3646

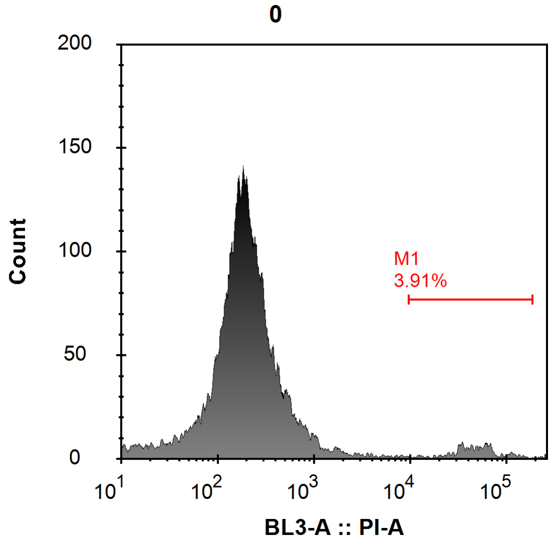

Gate	Count	%All	Mean X	Median X
All	13910	100.00%	2855	185
M1	544	3.91%	55186	47724

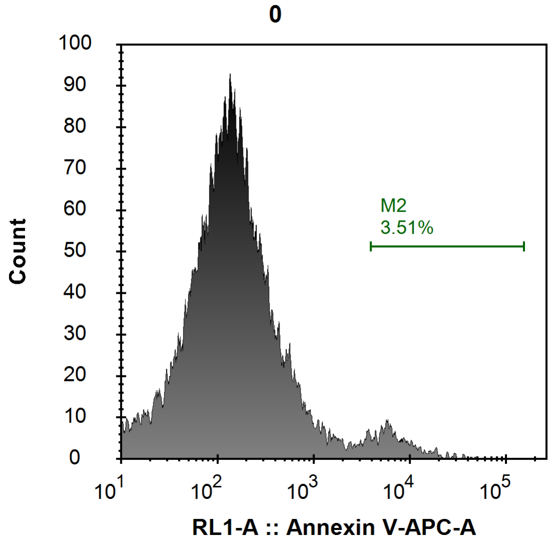

Gate	Count	%All	Mean X	Median X
All	13910	100.00%	556	122
M2	488	3.51%	9732	6953
