# Supplementary figures and images for: Lnc‐CHRM4‐2:1 Inhibits M2 Polarization and Efferocytosis of Macrophages by Downregulating MerTK and SLC2A1 in Rheumatoid Arthritis
Source: J Immunol Res. 2026 Feb 27;2026:1718207. doi: 10.1155/jimr/1718207 (PMC13140872; doi:10.1155/jimr/1718207)

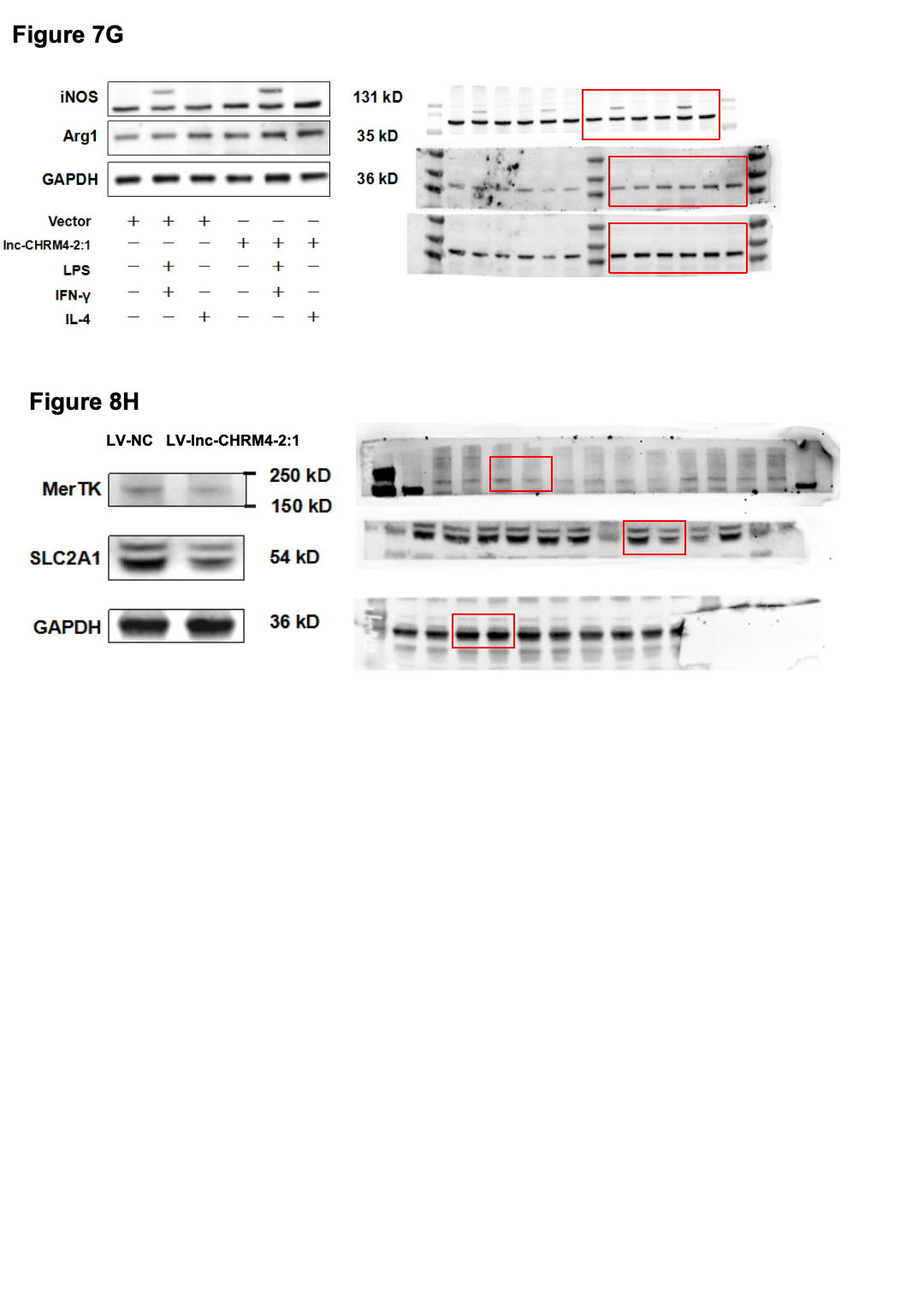

Supplement: Supplementary file 2 — Supporting Information 2 Figure 7G, Figure 8H .Raw Data_Western Blots.docx: original Western blot data and experimental parameters. All raw data match the experimental results in the main manuscript and support result validation. [file JIMR-2026-1718207-s001.docx]
